# Supplementary figures and images for: Low doses of diarrhoeagenic E. coli induce enhanced monocyte and mDC responses and prevent development of symptoms after homologous rechallenge
Source: PLoS One. 2023 Jan 6;18(1):e0279626. doi: 10.1371/journal.pone.0279626 (PMC9821474; doi:10.1371/journal.pone.0279626)

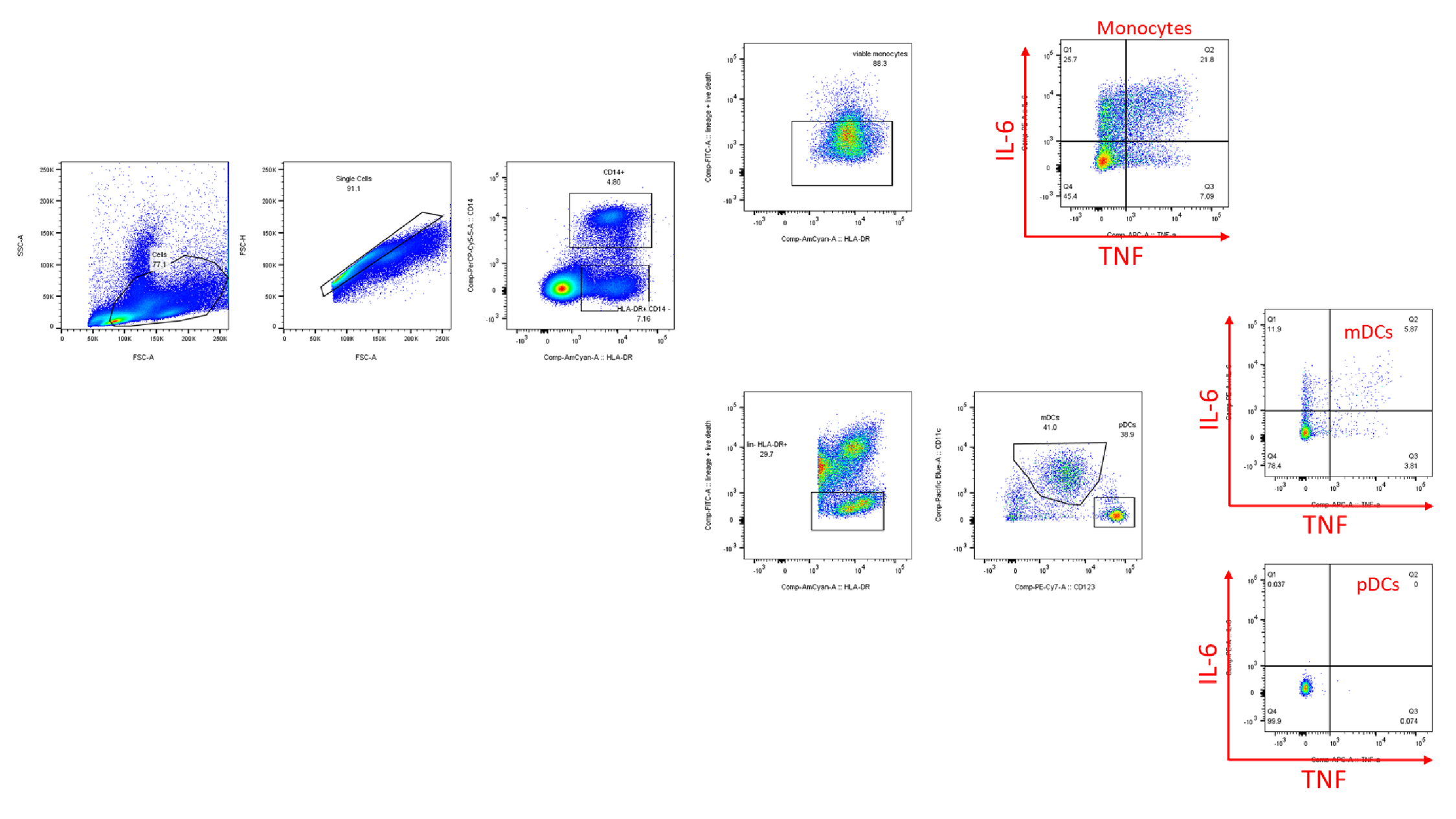

Supplement: S1 Fig — In the FSC/SSC plot cells within the PBMC region were selected. The duplets were gated out and the HLA-DR+CD14+ cells were considered as the monocytes. From the HLA-DR+CD14- population CD3+, CD19+, CD20+ and CD56+ cells were excluded. In the remaining population, CD11c+ cells were considered as mDCs and CD123+ cells were named pDCs. Within monocyte, mDC, and pDC populations cells that were producing IL-6, TNF-α or both cytokines were determined. (TIF) [file pone.0279626.s001.tif]

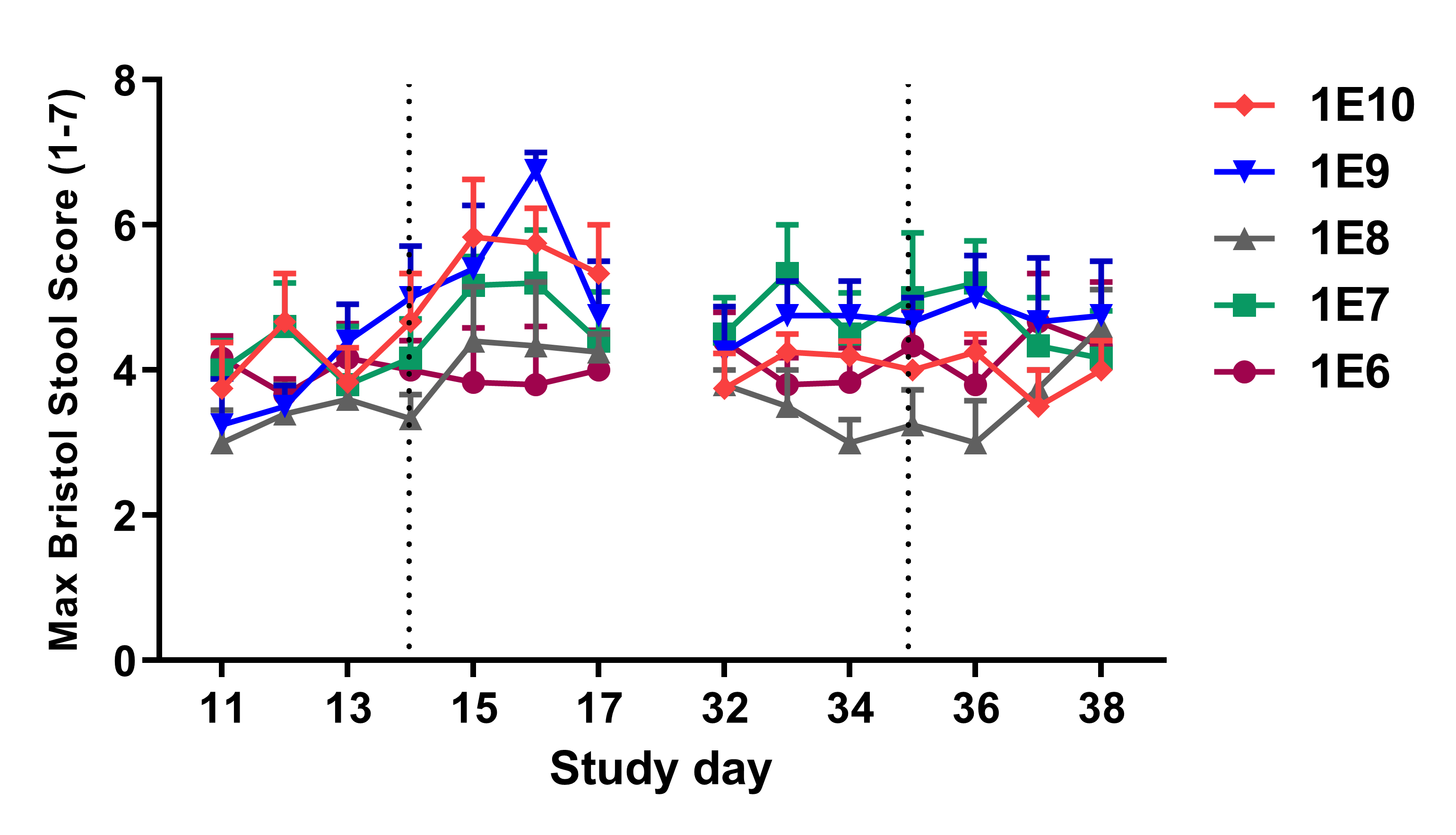

Supplement: S2 Fig — The changes in maximum Bristol stool score (stool consistency score) after primary infection or after reinfection were not dependent on the bacterial dose used during primary infection. The dotted lines represent infection days 14 and 35 of the study, and each symbol represents the mean and one-sided SEM of the group. Data were analyzed using repeated-measures Generalized Estimating Equations (GEE) model. (TIF) [file pone.0279626.s002.tif]

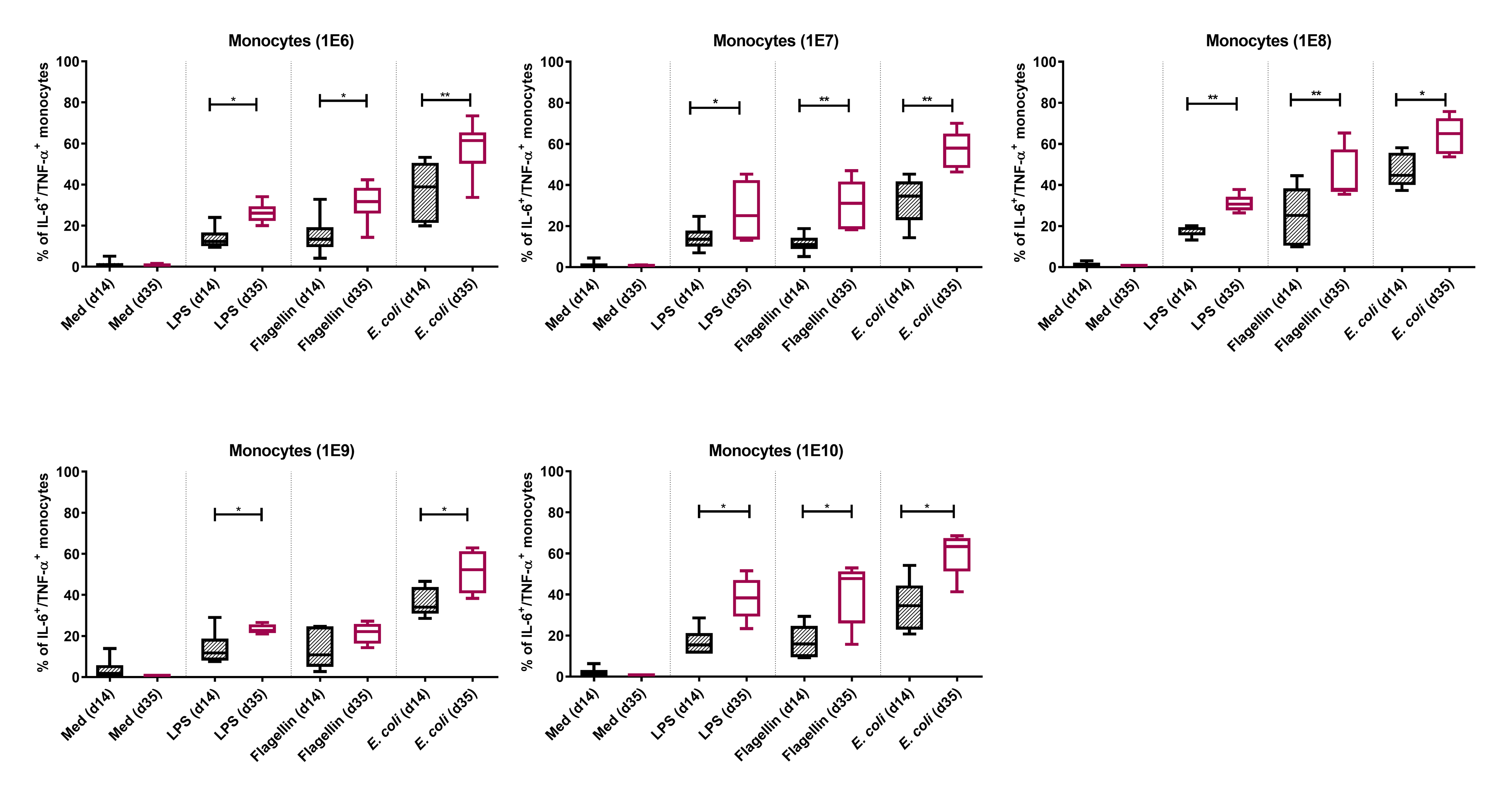

Supplement: S3 Fig — After ex vivo stimulation of PBMCs with either medium (RPMI-1640), 200 ng/mL of LPS, 500 ng/mL of Flagellin, or 1E7 CFU/well of E. coli (strain E1392/75-2A), the percentage of double-positive monocytes increased in all dose groups. This increase was significant after stimulation with flagellin and E. coli in group dose 1E6 and 1E7, flagellin in group dose 1E8, and following LPS, flagellin, ETEC stimulation in group 1E10. Data are shown in Whisker-plots with a median, 25% and 75% quartile. * p<0.05; ** p<0.01; *** p<0.001. (TIF) [file pone.0279626.s003.tif]

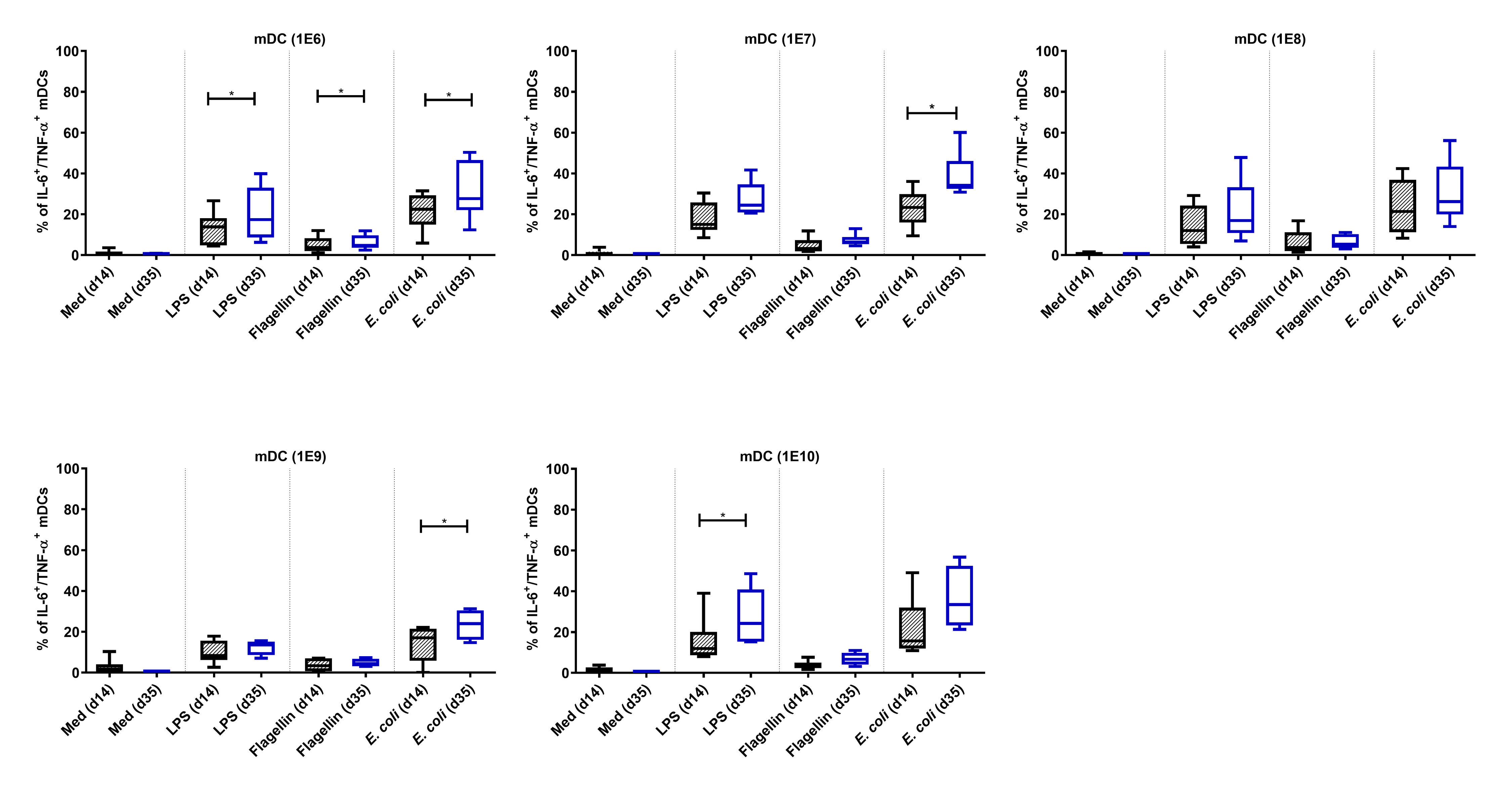

Supplement: S4 Fig — After ex vivo stimulation of PBMCs with either medium (RPMI-1640), 200 ng/mL of LPS, 500 ng/mL of Flagellin, or 1E7 CFU/well of E. coli (strain E1392/75-2A), the percentage of double-positive mDCs increased in all dose groups. This increase was significant after stimulation with E. coli in group dose 1E7. Data are shown in Whisker-plots with a median, 25% and 75% quartile. * p<0.05; ** p<0.01; *** p<0.001. (TIF) [file pone.0279626.s004.tif]

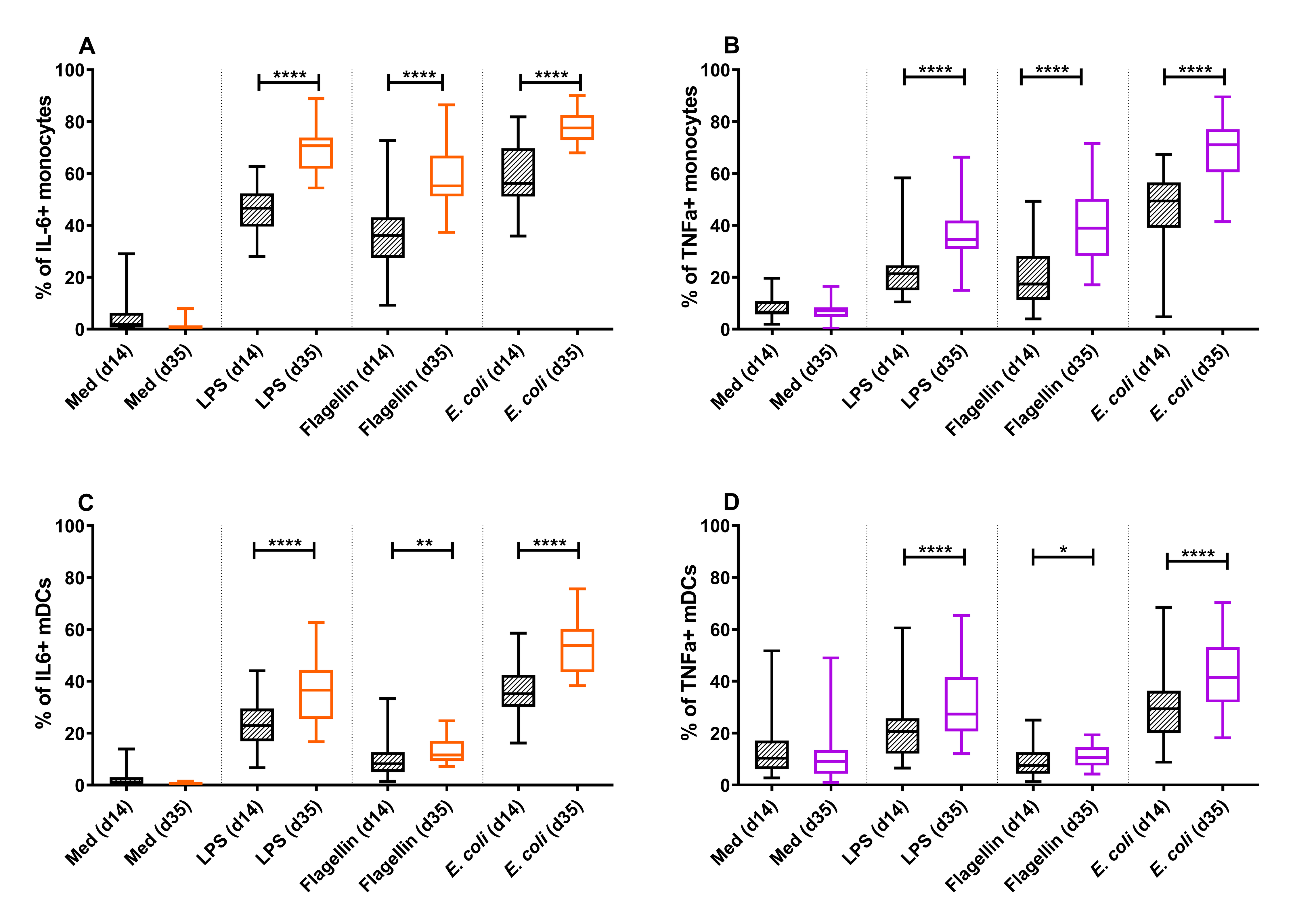

Supplement: S5 Fig — The cells were stimulated ex vivo with either medium (RPMI-1640), 200 ng/mL of LPS, 500 ng/mL of Flagellin, or 1E7 CFU/well of E. coli (strain E1392/75-2A). The percentage of IL-6+ as well as TNF-α+ monocytes increased significantly on day 35 compared to day 14. In mDCs, only LPS and E. coli increased the percentage of IL-6+ cells on day 35 and the significant increase in TNF-α+ mDCs only occurred after ETEC stimulation on day 35. Data are shown in Whisker-plots with a median, 25% and 75% quartile. * p<0.05; ** p<0.01; *** p<0.001. (TIF) [file pone.0279626.s005.tif]

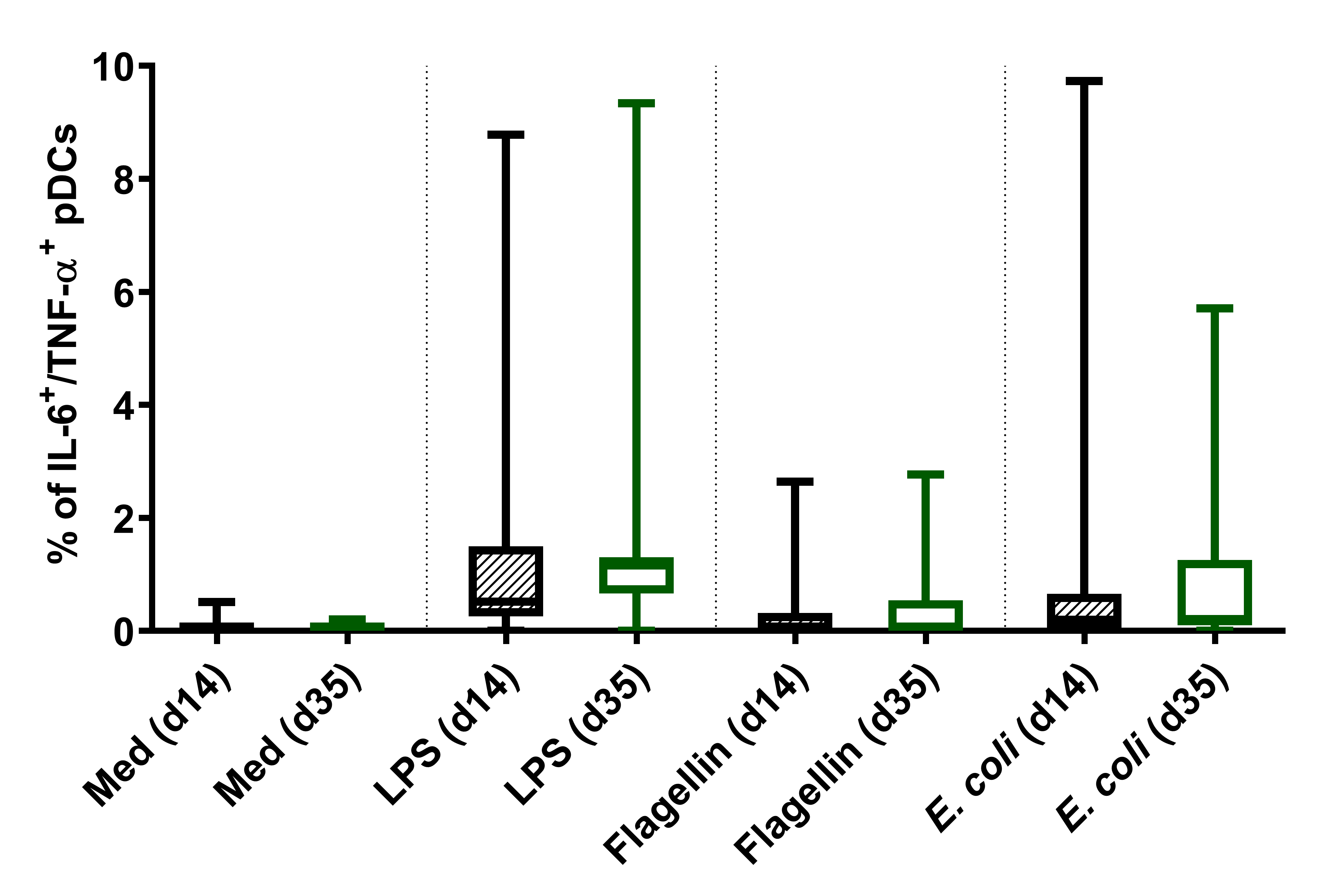

Supplement: S6 Fig — Ex vivo stimulation of pDCs with either medium (RPMI-1640), 200 ng/mL of LPS, 500 ng/mL of Flagellin, or 1E7 CFU/well of E. coli (strain E1392/75-2A), did not result in an increased percentage of double-positive pDCs. The Amount of IL-6 and TNF-α production in pDCs were marginal in all challenge doses and upon all different stimuli and were not significantly different between base line and day 21 of the study. (TIF) [file pone.0279626.s006.tif]
