# Supplementary material for: Low doses of diarrhoeagenic E. coli induce enhanced monocyte and mDC responses and prevent development of symptoms after homologous rechallenge
Source: PLoS One. 2023 Jan 6;18(1):e0279626. doi: 10.1371/journal.pone.0279626 (PMC9821474; doi:10.1371/journal.pone.0279626)
Supplement: S1 File — (PDF) [file pone.0279626.s008.pdf]

**RESEARCH PROTOCOL**

# MIRRE STUDY:
# THE PROTECTIVE IMMUNE RESPONSE TO ATTENUATED ENTEROTOXIGENIC ESCHERICHIA COLI INFECTION IN HEALTHY HUMAN SUBJECTS: A PILOT STUDY


**Research team**

| | | |
|---|---|---|
| Rianne Ruijschop PhD | Project manager | NIZO food research B.V. NL |
| Els van Hoffen PhD | Principal Investigator/ Deputy project manager | NIZO food research B.V. NL |
| Joyce Schloesser MSc | Study coordinator | NIZO food research B.V. NL |
| Elly Lucas | Scientific assistant | NIZO food research B.V. NL |
| Prof. Ben Witteman PhD | Gastroenterologist | Hospital Gelderse Vallei. NL |
| Prof. Joost van Neerven, PhD | Professor Mucosal Immunology, CBI | Wageningen University & Research |

# MIRRE STUDY: THE PROTECTIVE IMMUNE RESPONSE TO ATTENUATED ENTEROTOXIGENIC ESCHERICHIA COLI INFECTION IN HEALTHY HUMAN SUBJECTS: A PILOT STUDY

| | |
|---|---|
| **Protocol ID** | NL66039.028.18 |
| **Short title** | MIRRE |
| **EudraCT number** | *Not applicable* |
| **Version** | V1.1 |
| **Date** | 07-06-2018 |
| **Project manager**<br><br><br>**Study coordinator** | Rianne Ruijschop, PhD<br>T: +31(0)318 659 645<br>E: rianne.ruijschop@nizo.com<br><br>Joyce Schloesser, Msc<br>T: +31(0)318 659 563<br>E: joyce.schloesser@nizo.com<br><br>NIZO food research<br>Kernhemseweg 2<br>6718 ZB Ede<br>The Netherlands |
| **Principal investigator** | Els van Hoffen, PhD<br>T: +31(0)318 659 560<br>E: els.vanhoffen@nizo.com<br>NIZO food research<br>Kernhemseweg 2<br>6718 ZB Ede<br>The Netherlands |
| **Medical investigator** | Prof. Ben Witteman, PhD<br>Gelderse Vallei ziekenhuis<br>Ede, The Netherlands<br>T: +31 (0)318 435 390<br>E: wittemanb@zgv.nl |
| **Sponsor** | NIZO food research<br>Kernhemseweg 2<br>6718 ZB Ede<br>The Netherlands<br>T: +31 (0) 318 659 511 |
| **Independent expert** | Prof. Michiel Kleerebezem, PhD<br>Host-Microbe Interactomics<br>Wageningen University & Research<br>The Netherlands |

| | |
|---|---|
| | T: +31 (0) 317 486125<br>E: Michiel.Kleerebezem@wur.nl. |
| **Laboratory sites** | Elly Lucas<br>T: +31(0)318 659615<br>E: elly.lucas@nizo.com<br>NIZO food research<br>Kernhemseweg 2, 6718 ZB Ede<br><br>Prof. Joost van Neerven, PhD<br>Wageningen University & Research<br>Cell Biology and Immunology<br>Wageningen<br>The Netherlands<br>Tel (m): +31 (0)6 23537129<br>Email: joost.vanneerven@wur.nl |
| **Pharmacy** | *Not applicable* |

## PROTOCOL SIGNATURE SHEET

| Name | Signature | Date |
|---|---|---|
| **Sponsor or legal representative:**<br>Head of department:<br>Melle van Schaik<br>Division manager Health<br>Kernhemseweg 2<br>6718 ZB Ede<br>NIZO food research<br>The Netherlands | | |
| Principal Investigator:<br>Els van Hoffen, PhD<br>Senior scientist and Project manager Health<br>Kernhemseweg 2<br>6718 ZB Ede<br>NIZO food research<br>The Netherlands | | |

## TABLE OF CONTENTS

1. INTRODUCTION AND RATIONALE ....... 11
1.1 Food-borne infections ....... 11
1.2 Enterotoxigenic Escherichia coli ....... 11
1.3 Nutritional modulation of resistance to infection ....... 11
1.4 Diarrheagenic *E. coli* challenge model ....... 11
1.5 Adjustment of the diarrheagenic *E. coli* challenge model ....... 12
2. OBJECTIVES ....... 14
3. STUDY DESIGN ....... 15
4. STUDY POPULATION ....... 18
4.1 Population (base) ....... 18
4.2 Inclusion criteria ....... 18
4.3 Exclusion criteria ....... 19
4.4 Sample size calculation ....... 20
5. TREATMENT OF SUBJECTS ....... 21
5.1 Investigational treatment: Diarrheagenic *E. coli* inoculation ....... 21
5.2 Use of co-intervention ....... 21
6. INVESTIGATIONAL PRODUCT ....... 23
6.1 Name and description of investigational product ....... 23
6.2 Summary of findings from non-clinical studies ....... 23
6.3 Summary of findings from clinical studies ....... 25
6.4 Summary of known and potential risks and benefits ....... 28
6.5 Description and justification of route of administration and dosage ....... 29
6.6 Dosages, dosage modifications and method of administration ....... 29
6.7 Preparation and labelling of Investigational Product ....... 30
6.8 Investigational Product accountability ....... 30
7. NON-INVESTIGATIONAL PRODUCT ....... 31
8. METHODS ....... 32
8.1 Study parameters/endpoints ....... 32
8.1.1 Main study parameter/endpoint ....... 32
8.1.2 Secondary study parameters/endpoints ....... 32
8.1.3 Other study parameters ....... 32
8.2 Randomization, blinding and treatment allocation ....... 32
8.3 Study procedures ....... 33
8.3.1 Information meeting ....... 33
8.3.2 Pre-study screening; questionnaire ....... 33
8.3.3 Dietary restrictions ....... 33
8.3.4 Online diary and questionnaires ....... 34
8.3.5 Diarrheagenic *E. coli* inoculation ....... 34
8.3.6 Biological sample collection ....... 35
8.4 Withdrawal of individual subjects ....... 35
8.5 Replacement of individual subjects after withdrawal ....... 36

8.6 Follow-up of subjects withdrawn from treatment ........ 36
8.7 Premature termination of the study ........ 36
9. SAFETY REPORTING ........ 37
9.1 Temporary halt for reasons of subject safety ........ 37
9.2 AEs, SAEs and SUSARs ........ 37
9.2.1 Adverse events (AEs) ........ 37
9.2.2 Serious adverse events (SAEs) ........ 37
9.2.3 Suspected unexpected serious adverse reactions (SUSARs) ........ 38
9.3 Annual safety report ........ 38
9.4 Follow-up of adverse events ........ 38
9.5 Data Safety Monitoring Board (DSMB) ........ 38
10. STATISTICAL ANALYSIS ........ 40
10.1 Primary study parameter(s) ........ 41
10.2 Secondary study parameters ........ 41
10.3 Other study parameters ........ 42
10.4 Interim analysis ........ 43
11. ETHICAL CONSIDERATIONS ........ 44
11.1 Regulation statement ........ 44
11.2 Recruitment and consent ........ 44
11.3 Objection by minors or incapacitated subjects ........ 45
11.4 Benefits and risks assessment, group relatedness ........ 45
11.5 Compensation for injury ........ 46
11.6 Incentives (if applicable) ........ 46
12. ADMINISTRATIVE ASPECTS, MONITORING AND PUBLICATION ........ 48
12.1 Handling and storage of data and documents ........ 48
12.2 Monitoring and Quality Assurance ........ 48
12.3 Amendments ........ 48
12.4 Annual progress report ........ 49
12.5 Temporary halt and (prematurely) end of study report ........ 49
12.6 Public disclosure and publication policy ........ 49
13. STRUCTURED RISK ANALYSIS ........ 50
13.1 Potential issues of concern ........ 50
13.2 Synthesis ........ 52
14. REFERENCES ........ 52

## LIST OF ABBREVIATIONS AND RELEVANT DEFINITIONS

| | |
|---|---|
| **ABR** | ABR form, General Assessment and Registration form, is the application form that is required for submission to the accredited Ethics Committee (In Dutch, ABR = Algemene Beoordeling en Registratie) |
| **AE** | Adverse Event |
| **AR** | Adverse Reaction |
| **CCMO** | Central Committee on Research Involving Human Subjects; in Dutch: Centrale Commissie Mensgebonden Onderzoek |
| **CV** | Curriculum Vitae |
| **CFA** | Colonization Factor Antigen |
| **CFU** | Colony Forming Units |
| **CRF** | Case Report Form |
| **DSMB** | Data Safety Monitoring Board |
| **ETEC** | Enterotoxigenic *E. coli* |
| **GCP** | Good Clinical Practice |
| **IC** | Informed Consent |
| **LT** | Heat-Labile toxin |
| **METC** | Medical research ethics committee (MREC); in Dutch: medisch ethische toetsing commissie (METC) |
| **(S)AE** | (Serious) Adverse Event |
| **ST** | Heat-Stabile toxin |
| **Sponsor** | The sponsor is the party that commissions the organisation or performance of the research, for example a pharmaceutical company, academic hospital, scientific organisation or investigator. A party that provides funding for a study but does not commission it is not regarded as the sponsor, but referred to as a subsidising party. |
| **SUSAR** | Suspected Unexpected Serious Adverse Reaction |
| **Wbp** | Personal Data Protection Act (in Dutch: Wet Bescherming Persoonsgevens) |
| **WMO** | Medical Research Involving Human Subjects Act (in Dutch: Wet Medisch-wetenschappelijk Onderzoek met Mensen |

## SUMMARY

**Rationale:** The existing diarrheagenic *E. coli* challenge model is already suitable for dietary interventions targeted to impact on the immediate clinical symptoms upon *E. coli* infection. From previous studies we know that the current primary dose of infection ($1*10^{10}$ CFU) induces a maximum response, not only in the primary clinical response, but also immunologically as can be seen in protection against secondary infection. This results in induction of ETEC-specific antibody titers and absence of clinical symptoms upon a second *E. coli* inoculation. In order to make the model suitable for dietary interventions that are aimed at support of the protective response against reinfection, the immune response triggered by the primary infection should be suboptimal: antibody levels should not be at their maximum yet, and clinical symptoms should still be present when providing a second infection. It is expected that this can be achieved by lowering the primary infection dose. As it is not known which primary infection dose would be needed to achieve this, the aim of the MIRRE pilot study is to identify the infection dose at which the induced protective response at secondary infection is suboptimal.

**Objective**: Aim of the MIRRE pilot study is to determine how much the primary inoculation dose of diarrheagenic *E. coli* should be lowered in order to result in a reduced protective response upon a secondary infection.

**Study design:** The MIRRE pilot study is a parallel 7-weeks study. Subjects will be randomly assigned to one of five inoculation dosages of a live attenuated diarrheagenic *E. coli* strain (n=6 per group). Subjects will be instructed to maintain their usual pattern of physical activity and their habitual food intake, but to reduce and standardize their dietary calcium intake. After a standardized evening meal and an overnight fast, subjects will be orally infected on day 14 with a live, but attenuated, diarrheagenic *E. coli* (strain E1392/75-2A; collection NIZO food research). Five groups of 6 subjects will be provided one of the following dosages: $1*10^{10}$ CFU (standard dose); $1*10^{9}$ CFU; $1*10^{8}$ CFU; $1*10^{7}$ CFU; $1*10^{6}$ CFU. At study day 35, after a standardized evening meal and an overnight fast, all subjects will receive a second inoculation of $1*10^{10}$ CFU of the *E.coli* strain (n=30).
At various time points before and after both diarrheagenic *E. coli* infections, subjects will report information on stool consistency, frequency and severity of symptoms. Furthermore, at various time points before and after both *E. coli* infections, venous blood (3 times around first and 3 times around second infection) and 24h stool samples (4 days around first and 4 days around second infection) will be collected. Blood samples are collected to quantify antibody levels, and stool samples are collected to quantify fecal infection parameters.

**Study population:** Healthy male subjects, 18-55 years of age who fulfil all of the inclusion criteria and none of the exclusion criteria will participate in the MIRRE pilot study.

**Intervention**: At study day 14 and 35, after a standardized evening meal and an overnight fast, all subjects will receive an inoculation of the diarrheagenic *E. coli* strain ($1*10^{10}$ CFU (standard dose); $1*10^{9}$ CFU; $1*10^{8}$ CFU; $1*10^{7}$ CFU; $1*10^{6}$ CFU; n=6 per dose) at study day 14; $1*10^{10}$ CFU (n=30) at study day 35.
Subjects will be instructed to maintain their habitual diet, except for their dairy intake. Dairy has a high calcium content and contributes significantly to total daily calcium intake. These dietary guidelines will limit calcium intake on average to 500 mg/day. From previous studies performed by the sponsor, it is known that calcium can significantly reduce the gastro-intestinal symptoms induced by the *E. coli* strain.[1]

**Main study parameters/endpoints:**
Main study parameter:

- Specific antibody titer, serum IgG-CFA/II antibody levels, at second *E. coli* inoculation.

Secondary study parameters:

- Relative and total fecal wet weight (Freeze-drying of pooled 24 h fecal samples) at first and second *E. coli* inoculation
- Stool consistency (Bristol Stool Scale reported by the subjects in the online diary) at first and second *E. coli* inoculation
- Stool frequency (Stools per day reported by the subjects in the online diary) at first and second *E. coli* inoculation
- Incidence, duration and severity of Gastro-intestinal symptoms (Gastro-intestinal Symptom Rating Scale reported by the subjects in the online diary) at first and second *E. coli* inoculation

Tertiary exploratory study parameters:

- Functional immunological assays in peripheral blood mononuclear cells (response to TLR stimulation, phagocytosis, neutrophil function)
- Additional ELISA determinations to identify the response to infection (in plasma)
- Measurement of barrier/inflammation markers in fecal samples

The primary and secondary outcomes are aimed to obtain more insight into the clinical response to infection at five primary *E. coli* dosages, and the impact on the protective response at secondary infection. This dose-response information will help to establish the optimal design for a subsequent well-powered intervention study, aimed at support of the protective response at secondary infection.
The exploratory study parameters will help to identify additional correlates of protection against reinfection.

**Nature and extent of the burden and risks associated with participation, benefit and group relatedness:** *E. coli* strain E1392/75-2A is a live experimental oral vaccine, which does not produce toxins. Eight studies using this strain have been performed at NIZO between 2002 and 2017 (n=377 subjects). In general, during these infection studies, expected adverse events

are reported only the first 1-2 days after the diarrheagenic *E. coli* challenge, and are self-resolving. AEs include abdominal pain (~70-75% of subjects), abdominal distension (~50-55% of subjects), borborygmus (~65-70% of subjects), flatulence (~75-80 of subjects), increased passage of stools (~60-65% of subjects), loose stools (~60-65% of subjects), nausea (~55-60% of subjects), and urgent defecation (~55-60% of subjects). No serious adverse events related to the infection have been reported during the studies. The *E. coli* strain is sensitive to Ciprofloxacine, which is a commonly used antibiotic in case of treatment of this kind of *E. coli* infections, and can be used as rescue medication if needed. In all previous studies, all recorded disease episodes were self-limiting and did not require early antibiotic treatment.

# 1. INTRODUCTION AND RATIONALE

## 1.1 Food-borne infections

Food-borne infections occur frequently. However, the exact global incidence is difficult to estimate, because most food-borne infections are either not reported or not determined in biological samples. The WHO reported in 2007 that in industrialized countries, the percentage of the population suffering from food-borne diseases each year is up to 30%. This is probably an underestimation, since recent data from a Dutch study indicate that the incidence of infectious intestinal disease is 964 per 1000 person years.[2]

Food-borne infections are also frequently encountered by travelers to tropical countries, with incidences up to 80%. Travelers' diarrhea is the most common health impairment in persons visiting developing countries, affecting up to 50-90% of travelers in high risk areas.[3]

## 1.2 Enterotoxigenic Escherichia coli

Enterotoxigenic *Escherichia coli* (ETEC) is the leading bacterial cause of travelers' diarrhea.[4] ETEC infection is characterized by profuse and watery diarrhea lasting several days with abdominal cramp, malaise, vomiting and low grade fever.[5] Though it is rare in developed countries and usually benign, travelers' diarrhea represents a considerable socioeconomic burden for both the traveler and the host country.[3] For these reasons, much effort has been dedicated to finding a way of preventing such ailment.[4] Antibiotics can be a form of treatment, but the growing resistances of pathogens against antibiotics is a drawback. As a result, other forms of treating or preventing illness from food borne pathogens are being sought. Enhancement of human resistance to food-borne infections by functional food ingredients is therefore an attractive option.

## 1.3 Nutritional modulation of resistance to infection

Functional food ingredients can contribute to enhanced human resistance to infectious disease. The health benefits of functional food ingredients on defense against pathogens can for instance be studied in people who are travelling to a country of high risk for traveler's diarrhea, in other populations that are at risk for infections, like children in developing countries or in controlled infection challenge studies. The approach of the current setup allows to study the health benefits of functional food ingredients using a challenge study with a live, but attenuated, oral diarrheagenic *E.coli* strain, able to survive gastrointestinal transit and still able to induce mild (and short-lived) infection symptoms.

## 1.4 Diarrheagenic *E. coli* challenge model

Over the past 40 years, the enterotoxigenic *E. coli* (ETEC) human challenge model has been used to elucidate the pathogenesis and immune responses associated with ETEC infection as well as to test the efficacy of investigational drugs and vaccines. A systematic review of the published and unpublished literature to evaluate specific outcomes in subjects participating in experimental ETEC infection studies was published previously by Porter *et al* (2011).[6] Eleven

different strains were used in experimental challenge studies in human volunteers. Most commonly used were the toxin-producing strains B7A (O148:H28, CS6, LT/ST), E24377A (O139:H28, CS1/CS3, LT/ST), H10407 (O78:K80:H11, CFA/I, LT/ST).[6] The highest diarrhoea attack rates were seen with B7A at ~1E10 CFU (100.0%), H10407 at ~1E9 CFU (87.5%) and E24377A at ~5E9 CFU (80.8%). Strains expressing CS17 (LSN03-016011/A; LT only) and CS19 (WS0115A; LT&ST) also caused diarrhea, although at lower attack rates. Another strain expressing CS19 (DS26-1) caused no diarrhea.[6]

The basic concept of the diarrheagenic *E.coli* strain challenge study that NIZO developed is based on selection of a well-characterized, antibiotic susceptible organism that has been associated with mild diarrhea and gastrointestinal symptoms (severity and duration).[1, 7, 8] In all previous studies, recorded disease episodes were self-limiting and did not require early antibiotic treatment. The strain used at NIZO food research, the diarrheagenic *E.coli* strain E1392/75-2A, is a spontaneous mutant with deletion of the genes encoding the LT and ST toxins, and can therefore not produce any toxins. However, it continues to express CFA/II and provides 75% protection against challenge with an LT, ST, CFA/II strain.[5]

### 1.5 Adjustment of the diarrheagenic *E. coli* challenge model

In a previous study (CORAL; https://clinicaltrials.gov/ct2/show/NCT02541695) the administration of a second inoculation of the attenuated diarrheagenic *E. coli* on day 35 was introduced. This second inoculation can be used to assess whether the primary infection is able to induce a protective response against secondary infection. A study by Harro *et al* clearly demonstrated that subjects who experienced ETEC-induced diarrhea in the study with ETEC H10407 were protected clinically when they were exposed to a second challenge with the same strain.[9] Our previous CORAL study also showed that the standard dose of *E. coli* that is used for primary infection ($1*10^{10}$ CFU) induces a clear antibody response at the time of the secondary infection that does not further increase upon secondary infection, and that clinical symptoms at secondary infection are mostly absent, suggesting that the primary infection induced a maximum protective response. This maximum protection is a disadvantage for (dietary) interventions aimed at the improvement of the protective response that is induced, as further support of the protective is not possible. Therefore, the model needs adjustment in order to reduce the protective (immune) response against secondary infection induced by the primary infection. Most likely, lowering the primary infection dose will reduce the antibody level that is induced, and thereby reduce the protective response, still resulting in clinical symptoms, upon secondary infection. Studies using wild-type *E. coli* strains have indeed shown that lower inoculation dosages may reduce the acute clinical symptoms as well as the antibody response induced after primary infection, as reviewed by Porter et al.[6] Various strains and dosages ranging from $1*10^6$ to $1*10^{10}$ have been used in other studies. However, as the NIZO strain is an attenuated strain, and different strains may behave differently in a dose-response analysis, it is not feasible to select the exact lower dose needed to induce a suboptimal antibody response, and to have remaining symptoms upon secondary infection. In order to reduce the risk of failures in subsequent intervention studies, the MIRRE pilot study

is aimed to get a better preliminary understanding of the dose-response of the attenuated *E. coli* strain. The fact that the study is designed as a pilot study means that no significant effects are expected. However, by using 5 different primary infection dosages, a dose-response effect is expected, of which the direction and the variation of the results will provide relevant information about the impact of primary infection dose on the response to the secondary infection. This information will be used to establish the correct design of subsequent adequately-powered dietary intervention studies aimed at support of the secondary protective response.

## 2. OBJECTIVES

To investigate whether the experimental challenge with the infectious diarrhea-inducing attenuated diarrheagenic *Escherichia coli* model can be “tuned” to predominantly focus on the host immune response.

The hypothesis of this pilot study is that the *E. coli* infection dose at primary inoculation determines the outcomes of the protective immune response and the extent of clinical symptoms at a secondary inoculation. More specifically, it is hypothesized that the level of CFA/II-specific IgG antibodies induced after the primary infection is inversely related to the severity of clinical symptoms as observed at the secondary infection. Therefore, in this pilot study, the CFA/II-specific IgG antibody response, in relation to clinical symptoms, is the main outcome. If this inverse relationship is confirmed, the optimal primary inoculation dose can be selected that is needed to provide suboptimal protection at the secondary inoculation.
Furthermore, by also studying additional immune parameters in a more explorative setting, valuable information will be gained to understand the mechanism of action of the model.

The main study parameter is:

- Specific antibody titer, serum IgG-CFA/II.

Secondary study parameters are:

- Relative and total fecal wet weight (Freeze-drying of pooled 24 h fecal samples) at first and second E. coli inoculation)
- Stool consistency (Bristol Stool Scale reported by the subjects in the online diary) at first and second E. coli inoculation
- Stool frequency (Stools per day reported by the subjects in the online diary) at first and second E. coli inoculation
- Incidence, duration and severity of Gastro-intestinal symptoms (Gastro-intestinal Symptom Rating Scale reported by the subjects in the online diary) at first and second E. coli inoculation.

Tertiary exploratory study parameters:

- Functional immunological assays in peripheral blood mononuclear cells (response to TLR stimulation, phagocytosis, neutrophil function)
- Additional ELISA determinations to identify the response to infection (in plasma)
- Measurement of barrier/inflammation markers in fecal samples

## 3. STUDY DESIGN

The MIRRE pilot study is a randomized, double-blind dose-response, parallel human infection study in 30 healthy adults (see study schedule below). The study will include 5 dosages (see table below). All subjects will start the study simultaneously.

Schematic presentation of the MIRRE pilot study

| **PERIOD I** | | | | | | | | | | | | | | | |
|---|---|---|---|---|---|---|---|---|---|---|---|---|---|---|---|
| **Activities** | | 1-10 | 11 | 12 | 13 | 14 | 15 | 16 | 17 | 18 | 19 | 20 | 21 | 22-27 | 28 |
| | | | | | | | | | | | | | | | |
| Informed consent & Screening | | | | | | | | | | | | | | | |
| Restricted intake specific medicine | | | | | | | | | | | | | | | |
| Restricted alcohol intake | | | | | | | | | | | | | | | |
| Standardized evening meal | | | | | | | | | | | | | | | |
| Overnight fast | | | | | | | | | | | | | | | |
| Infection attenuated E. coli | | | | | | | | | | | | | | | |
| Data Safety Monitoring Board | | | | | | | | | | | | | | | |
| Collection 24h fecal samples | | | | | | | | | | | | | | | |
| Collection blood sample | | | | | | | | | | | | | | | |
| Bristol stool scale (online) | | | | | | | | | | | | | | | |
| Stool frequency (online) | | | | | | | | | | | | | | | |
| GSRS (online) | | | | | | | | | | | | | | | |
| Restricted dairy intake & dietary guidelines | | | | | | | | | | | | | | | |
| Registration medication intake & compliance (online) | | | | | | | | | | | | | | | |

| **PERIOD II** | | | | | | | | | | | | | | | |
|---|---|---|---|---|---|---|---|---|---|---|---|---|---|---|---|
| **Activities** | | 29-31 | 32 | 33 | 34 | 35 | 36 | 37 | 38 | 39 | 40 | 41 | 42 | 43-48 | 49 |
| | | | | | | | | | | | | | | | |
| Restricted intake specific medicine | | | | | | | | | | | | | | | |
| Restricted alcohol intake | | | | | | | | | | | | | | | |
| Standardized evening meal | | | | | | | | | | | | | | | |
| Overnight fast | | | | | | | | | | | | | | | |
| Infection attenuated E. coli | | | | | | | | | | | | | | | |
| Data Safety Monitoring Board | | | | | | | | | | | | | | | |
| Collection 24h fecal samples | | | | | | | | | | | | | | | |
| Collection blood sample | | | | | | | | | | | | | | | |
| Bristol stool scale (online) | | | | | | | | | | | | | | | |
| Stool frequency (online) | | | | | | | | | | | | | | | |
| GSRS (online) | | | | | | | | | | | | | | | |
| Restricted dairy intake & dietary guidelines | | | | | | | | | | | | | | | |
| Registration medication intake & compliance (online) | | | | | | | | | | | | | | | |

**Selected dosages in the MIRRE pilot study**

| Study arm | Description | Relevant comparisons |
|---|---|---|
| 1 | First inoculation: $1*10^{10}$, Second inoculation $1*10^{10}$, low-calcium diet | |
| 2 | First inoculation: $1*10^{9}$, Second inoculation $1*10^{10}$, low-calcium diet | Compared to 1 (effect E. coli dose) |
| 3 | First inoculation: $1*10^{8}$, Second inoculation $1*10^{10}$, low-calcium diet | Compared to 1 (effect E. coli dose) |
| 4 | First inoculation: $1*10^{7}$, Second inoculation $1*10^{10}$, low-calcium diet | Compared to 1 (effect E. coli dose) |
| 5 | First inoculation: $1*10^{6}$, Second inoculation $1*10^{10}$, low-calcium diet | Compared to 1 (effect E. coli dose) |

30 healthy human subjects recruited from the Wageningen/Ede (The Netherlands) area who fulfill all of the inclusion criteria and none of the exclusion criteria will participate in the MIRRE pilot study. Subjects will be stratified according to Age and BMI.

Subjects will be instructed to maintain their usual pattern of physical activity and their habitual food intake, but to standardize their dietary calcium intake. The dietary guidelines will be stated in the subject diary and will limit calcium intake on average to 500 mg/day. Dairy has a high calcium content and contributes significantly to total daily calcium intake. From our previous studies, we know that calcium can significantly reduce the gastro-intestinal symptoms induced by the ETEC strain.[1]
After an overnight fast, subjects will be orally infected with a live, but attenuated, diarrheagenic *E. coli* (strain E1392/75-2A; collection NIZO food research) (at study day 14 and 35).

Use of prescription and over-the-counter (OTC) medications that contain non-steroidal anti-inflammatory drugs, acid suppression medication, antimotility and any pre-/probiotics and antibiotics are prohibited on the three days before, during and after the diarrheagenic *E. coli* challenge. Paracetamol up to 2 g/day is allowed as escape medication during these days. Subjects will receive a list of prohibited and permissible medication. However, if medication is prescribed by the general practitioner or study physician, subjects are allowed to take these medications but need to register intake (dose and frequency). If medically safe, subjects will stay in the study.

Throughout the entire study, subjects will be asked to indicate compliance to study guidelines in an online diary. Before and after the diarrheagenic *E. coli* challenge an online diary will be kept to record information on stool consistency, frequency, severity of symptoms and

mediation intake. At various time points before and after diarrheagenic *E. coli* challenge blood and stool samples will be collected.

# 4. STUDY POPULATION

## 4.1 Population (base)

30 healthy male subjects recruited from the Wageningen/Ede (The Netherlands) area who fulfill all of the inclusion criteria and none of the exclusion criteria (stated below). Subjects will be stratified according to age and BMI.
Women are excluded from participation because of the influence of the menstrual cycle on gut comfort and reported symptoms. It is also more difficult for women to collect urine and feces separately.

## 4.2 Inclusion criteria

In order to be eligible to participate in this study, a subject must meet all of the following criteria:

Substantial:
1. Male
2. Age between 18 and 55 years.
3. BMI ≥18.5 and ≤30.0 kg/m2.
4. Healthy as assessed by the NIZO health questionnaire.

Procedural:
5. Ability to follow Dutch verbal and written instructions.
6. Availability of internet connection.
7. Signed informed consent.
8. Willing to accept disclosure of the financial benefit of participation in the study to the authorities concerned.
9. Willing to accept use of all encoded data, including publication, and the confidential use and storage of all data for at least 15 years.
10. Willing to comply with study procedures, including collection of stool and blood samples.
11. Willingness to abstain from high calcium containing products.
12. Willingness to abstain from alcoholic beverages three days before, during and for 4 days after diarrheagenic *E. coli* challenge.
13. Willingness to abstain from medications that contain acetaminophen, aspirin, ibuprofen, and other non-steroidal anti-inflammatory drugs, (OTC) antacids and antimotility agents (eg, loperamide) on the three days before, during and for 4 days after diarrheagenic *E. coli* challenge.
14. Willingness to abstain from probiotics and prebiotic/fibers starting from runin and during the whole study.
15. Willingness to give up blood donation starting at run-in and during the entire study.

## 4.3 Exclusion criteria

A potential subject who meets any of the following criteria will be excluded from participation in this study:

Substantial*:

1. Acute gastroenteritis in the 2 months prior to inclusion
2. Any confirmed or suspected immunosuppressive or immunodeficient condition including human immunodeficiency virus infection (HIV).
3. Disease of the GI tract, liver, gall bladder, kidney, thyroid gland (self-reported), except for appendicitis.
4. History of microbiologically confirmed ETEC or cholera infection within 3 years prior to inclusion.
5. Symptoms consistent with Travelers' Diarrhea concurrent with travel to countries where ETEC infection is endemic (most of the developing world) within 3 years prior to inclusion, OR planned travel to endemic countries during the length of the study.
6. Vaccination for, or ingestion of cholera within 3 years prior to inclusion, including studies at NIZO.
7. Occupation involving handling of ETEC or Vibrio cholerae currently, or within 3 years prior to inclusion.
8. Vaccination for, or ingestion of ETEC or *E coli* heat labile toxin, including *E. coli* challenge studies at NIZO.
9. Evidence of current excessive alcohol consumption (>4 consumptions/day or >20 consumptions/week) or drug (ab)use, and not willing/able to stop this during the study.
10. Known allergy to the following antibiotics: ciprofloxacin, trimethoprim, sulfamethoxazole, and penicillins.
11. Reported average stool frequency of >3 per day or <1 per 2 days.
12. Use of antibiotics, (during 6 months prior to inclusion), norit, laxatives, cholestyramine, antacids H2 receptor antagonists or proton pump inhibitors (during 3 months prior to inclusion).
13. Use of immunosuppressive drugs (e.g. cyclosporine, azathioprine, systemic corticosteroids, antibodies).
14. Vegans.
15. Mental status that is incompatible with the proper conduct of the study.

Procedural:

1. Not having a general practitioner, not allowing disclosure of participation to the general practitioner or not allowing to inform the general practitioner about abnormal results.
2. Participation in any clinical trial including blood sampling and/or administration of substances starting 1 month prior to study start and during the entire study.
3. Personnel that is part of the study team at NIZO, their partner and their first and second degree relatives.

* Please note: in this pilot study, absence of carriage of colonization factor antigen type II (CFA/II)-positive bacteria (mainly endogenous *E. coli* strains) in a fecal sample, or presence of CFA/II-specific IgG antibodies in serum, collected at screening before the study starts, will not be analyzed. Previous experience shows that this may lead to 1-2 exclusions, which does not outweigh the extra effort and costs of measuring that on forehand.

### 4.4 Sample size calculation

In the current experiment an evaluation is performed to detect a potential association between the dose of the infective microorganism provided at the first inoculation and a reduced protective response at a second. Therefore 5 doses have been selected without a negative control. According to the protocol of Plackett and Burman[10] (Design of Experiment: DOE) the highest and the lowest dose should be studied for efficacy while the intermediate doses will be used for variation information. Thereby the relationship of the effect and dosing association could be determined within the interval as used in the present study. However, in a typical Plackett-Burman design 4 data points are required to estimate the variance, especially when no negative control (no dose at all) has been implemented. This is done to gain information about inter- and intra-variation of the doses provided with respect to the efficacy obtained. Background information would have been available when a dose of no microorganisms was administered. To compensate for this, per dose of microorganisms an additional person is added. This adds up to 6 persons per dose, allowing evaluation of normality per dose used.

# 5. TREATMENT OF SUBJECTS

## 5.1 Investigational treatment: Diarrheagenic *E. coli* inoculation

After a runin period of 13 days, on day 13 a standardized evening meal will be provided to the subjects (see study scheme paragraph 3). At study day 14, after an overnight fast, subjects will receive a single oral dose of the attenuated diarrheagenic *E. coli* strain E1392-75-2A (dose will be either $1*10^{10}$ CFU (n=6), $1*10^{9}$ CFU (n=6), $1*10^{8}$ CFU (n=6), $1*10^{7}$ CFU (n=6), or $1*10^{6}$ CFU (n=6). Oral challenge will occur at 10.00 AM. Under supervision of the project team, subjects will get a $NaHCO_3$ solution (100 mL 2% $NaHCO_3$) to neutralize the gastric acid. After 5 minutes, they get a fruit juice (100 mL) containing the attenuated diarrheagenic *E. coli* strain at the above-mentioned dose. Subjects go home, but are not allowed to drink or eat for 1 hour.

At study day 35, after a standardized evening meal and an overnight fast, all subjects will receive a second inoculation $1*10^{10}$ CFU of the diarrheagenic *E. coli*. E1392/75-2A (all at a dose: $1*10^{10}$ CFU). Oral challenge will occur according to the same procedure as described for day 14.

## 5.2 Use of co-intervention

*Dietary calcium restriction*

Subjects will be instructed to maintain their habitual diet, except for their dairy intake. Dairy has a high calcium content and contributes significantly to total daily calcium intake. These dietary guidelines will limit calcium intake on average to 500 mg/day. From our previous studies, we know that calcium can significantly reduce the gastro-intestinal symptoms induced by the ETEC strain[5].

In general, Dutch people have a high dairy and thus dietary calcium intake in comparison with many other countries. As dietary calcium affects the resistance of subjects to intestinal infections and also affects the efficacy of the oral vaccine [5], their dietary calcium intake during the study has to be lowered temporarily. Subjects will be asked to maintain their habitual diet but to omit the following dairy products:

- Milk (all kinds, except for small amounts of coffee milk or creamer)
- Yogurt (all kinds)
- Custard (all kinds)
- Buttermilk (all kinds)
- Curds
- Cheese (all kinds)

It is estimated that this restriction in dairy intake will lower subject's daily calcium intake to 500-700 mg.[1] This intake is below the recommended daily calcium intake of 1000 mg in The Netherlands, but is quite common in very large population groups around the world (e.g. in Southern Europe, Asian countries, African-Americans, elderly, lactose-intolerant subjects). This temporary restriction in dietary calcium intake is not considered harmful.

*Probiotics and prebiotics/fibers*

Based on published study results and published literature(s), it has been observed that use of probiotics, probiotic based products or prebiotics/fibers during diarrhea episode may affect the duration of the diarrhea. Hence, participating subjects will be instructed to avoid probiotics or probiotic based products and prebiotic/fiber supplements, starting from the run-in period and during the whole study. Subject will be asked to omit below products in general:

- Probiotics (all kinds)
- Non-dairy Yoghurts with active cultures of probiotics
- Probiotic based supplements
- Products with added prebiotics/fibers
- Prebiotic/fiber supplements

*Escape medication*

Paracetamol up to 2 g/day is allowed as escape medication during these days.

# 6. INVESTIGATIONAL PRODUCT

## 6.1 Name and description of investigational product

Diarrheagenic *E. coli* strain E1392/75-2A serotype O6:H16 (supplier: Acambis, Cambridge, UK) belongs to Pathogen class 2. The strain has a spontaneous deletion of the genes encoding the LT and ST toxins and can therefore not produce any toxins. However, it continues to express CFA/II and provides 75% protection against challenge with an LT, ST, CFA/II strain. Therefore, this strain is a so-called live attenuated strain: the strain can still grow, but induces only mild and transient disease.

Although the strain lacks enterotoxin genes, the genome sequence of the attenuated diarrheagenic *E. coli* strain (E1392-75-2A) indicates that it resembles the following strains H10407, UMEA3212-1 and KTE103. The strain E1392-75-2A still has many genes coding for "colonization factors" and elements for excretion of potential toxins.

## 6.2 Summary of findings from non-clinical studies

NIZO food research has previously performed 4 nutritional intervention studies in animals with diarrheagenic *E. coli* strain E1392/75(2A) (see Table below).

| **Animal studies using Diarrheagenic *E. coli* strain E1392/75-2A Study or NIZO Report Number** | **Author(s)** | **Title of report/publication** |
|---|---|---|
| **Scientific publication** | Bovee *et al* | Diarrhea Caused by Enterotoxigenic Escherichia coli Infection of Humans Is Inhibited by Dietary Calcium[1] |
| **E2010/048** | Ten Bruggencate *et al.* | Dairy ingredients and the resistance to enteric infections induced by *Salmonella enteritidis or* Enterotoxigenic *escherichia coli.* |
| **E2010/110** | Ten Bruggencate *et al.* | Lactoferrin and resistance to Enterotoxigenic *E. coli*-induced gastroenteritis |
| **E 2011/171** | Sprong *et al.* | Human milk oligosaccharides and resistance to enterotoxigenic *E. coli* infection in rats. |

In the first study by Bovee *et al*, male Wistar rats (n=12 rats per dietary group) were housed and fed were fed diets containing low-calcium (negative control) or high-calcium (positive control).[1] Calcium contents of the diets were 0.8 and 4 mg/kg (20 and 100 mmol/kg) diet. After an adaptation period of 2 weeks, $10^9$ colony-forming units (CFU) diarrheagenic *E. coli* strain E1392/75-2A was orally administered. After several days, fresh fecal samples were collected and analyzed for the number of viable *E. coli* by plating. In addition, 24-hour feces was analyzed for cations to determine fecal water content.
Average food consumption (18 g/day) and body weight gain (3.5 g/day) of the rats were not significantly affected by calcium or diarrheagenic *E. coli*. Dietary calcium notably decreased intestinal colonization of diarrheagenic *E. coli* in rats as determined by the significantly reduced fecal shedding of this pathogen with time. Concomitantly, diarrheagenic *E. coli* infection increased the concentration of fecal cations and thus increased the relative water content of rat feces. Calcium largely prevented the diarrheagenic *E. coli* -induced increase in fecal cation concentration.

In the second study by Ten Bruggencate *et al* (E2010/048) male Wistar rats (n=8 rats per dietary group), received low or high-calcium with/without various dairy ingredients. After adaptation to the housing and dietary conditions for 14 days, rats were orally infected by gastric gavage of $3*10^8$ colony-forming units (CFU) of *S. enteritidis* or $6*10^9$ CFU diarrheagenic *E. coli.* Animals were sacrificed 9 days after infection. The severity of the infection was determined by quantifying animal growth and feed and energy intake. Furthermore, diarrhea was determined as a marker for disease (by measuring fecal wet and dry weight). The colonization of the pathogens was evaluated by determining excretion of *S. enteritidis* and diarrheagenic *E. coli* in feces by plating on specific culture media, at different time points after infection.
During the first week after *S. enteritidis* and ETEC infection, average energy intake decreased. After infection with either *S. enteritidis* or diarrheagenic *E. coli*, no infection-induced wasting was apparent in any of the dietary groups. diarrheagenic *E. coli* increased infectious diarrhea was determined by calculating the percentage of fecal dry weight. Fecal diarrheagenic *E. coli* counts were $10^8$ CFU/g feces at day 1 after challenge. At day 6 after challenge, diarrheagenic *E. coli* counts were below detection limit in the high-calcium group, while the low-calcium group still had detectable counts ($10^4$-$10^5$ CFU/g feces).

In the third study by Ten Bruggencate *et al* (E2010/110), male Wistar rats (n=16 rats per dietary group) were fed diets containing low-calcium (negative control) or high-calcium (positive control) and low or high lactoferrin. The negative control diet contained 30 mmol/kg calcium, while the positive control diet contained 100 mmol calcium/kg. After 14 days, rats were orally infected by gastric gavage of $1*10^{10}$ colony-forming units (CFU) of diarrheagenic *E. coli* strain E1392/75-2A. Basic well-being of the animals was determined by monitoring animal growth, feed intake and infectious diarrhea. Colonization and mucosal adhesion of diarrheagenic *E. coli* were determined as parameters for pathogen persistence.

During the first week after challenge with the diarrheagenic *E. coli* strain, average food intake decreased in the low-calcium group. No decrease in food intake was observed in the high-calcium group. As expected, after infection with diarrheagenic *E. coli* strain, no infection-induced wasting was apparent in any of the dietary groups. Fecal diarrheagenic *E. coli* counts were $10^8$-$10^9$ CFU/g feces at day 1 after challenge. Dietary calcium notably decreased intestinal colonization of diarrheagenic *E. coli* in rats as determined by the significantly reduced fecal shedding of this pathogen with time (10-10,000 fold). At day 6 after challenge, diarrheagenic *E. coli* counts were below detection limit in the high-calcium group, while the low-calcium group still had detectable counts ($10^7$ CFU/g feces). In the duodenum, no *E. coli* was detected in any of the dietary groups. In contrast, diarrheagenic *E. coli* was found in ileal mucosal samples ($10^2$-$10^3$ CFU/g ileal mucosa). Infectious diarrhea was determined by calculating the percentage of fecal dry weight. Diarrheagenic *E. coli* slightly decreases the percentage of fecal dry weight measured at day 2 after challenge but levels were back to normal at day 4 after challenge.

In the fourth study (E2011/171) by Sprong *et al*, male Wistar rats (n=8 rats per dietary group) were fed diets containing low or high calcium with/without human milk oligosaccharides. After 14 days, rats were orally infected by gastric gavage of $1*10^9$ colony-forming units (CFU) of diarrheagenic *E. coli*. Basic well-being of the animals was determined by monitoring growth and feed intake. Colonization by diarrheagenic *E. coli* and infectious diarrhea were determined as parameters for pathogen persistence. Intestinal permeability was determined by measuring urinary CrEDTA and lactulose/mannitol excretion.
All dietary groups showed a drop in feed intake after inoculation with diarrheagenic *E. coli*, but this was normalized within two days after diarrheagenic *E. coli* infection. The drop in feed intake coincided with a decrease in bodyweight gain just after diarrheagenic *E. coli* infection. Whole gut permeability was increased on day 2 of infection for all groups. The calcium group showed significantly less whole gut permeability compared to the control group at all post-infection time-points. Diarrheagenic *E. coli* transiently increased transcellular permeability but it returned to baseline levels at day 4 of infection. After infection, diarrheagenic *E. coli* -induced mannitol permeability was lower in the calcium group at day 2 and day 6.

### 6.3 Summary of findings from clinical studies

The *E. coli* strain E1392/75(2A) is used in several studies described by the group of Levine,[5, 11] and is given by them in doses up to $6*10^{10}$ organisms per subject. Vaccination experiments with this ETEC strain in humans are published by e.g. Tacket *et al*. In their study, after oral administration of $1*10^{10}$ CFU, 15% of the vaccinated persons suffered from self-limited, mild diarrhea with spontaneous recovery after 1-3 days.[5]
NIZO food research has previously performed 8 nutritional intervention studies (n=377) in humans with *E. coli* strain E1392/75(2A) (see Table below).
In these studies, the diarrheagenic *E. coli* strain transiently induces symptoms of a food borne infection increasing infectious diarrhea, fecal pathogen excretion, stool frequency, Bristol

Stool Score, reported symptoms, secretory IgA, C-reactive protein, calprotectin and antibody responses.[1, 7, 12] The diarrheagenic *E. coli* strain induces a small increase in average stool frequency from 1 stool/day to 2 stools/day, and an increase in average daily Bristol Stool Score from 4 to 5. Types 1–2 on the Bristol Stool Scale indicate constipation, with 3 and 4 being the ideal stools (as they are easy to defecate while not containing any excess liquid), and 5-7 tending towards diarrhea. All recorded disease episodes were self-limiting and did not require early antibiotic treatment. No treatment-related serious adverse events were reported during these studies. Results from the primary outcomes in these studies (infectious diarrhea as determined by fecal wet weight (fecal output) or percentage of fecal dry weight (stool consistency)) are presented below.

| **Clinical studies using diarrheagenic *E. coli* strain E1392/75-2A Study acronym** | **Ingredient** | **Year** | **Subjects** | **ETEC dose (CFU)** | **Clinical Trials Registration** | **Scientific publication reference** |
|---|---|---|---|---|---|---|
| **CARE** | Milk calcium | 2002 | 32 | $1*10^{10}$ | Not submitted | [1] |
| **PROLARE** | Probiotics | 2006 | 32 | $6*10^{9}$ | Not submitted | |
| **PRETEC** | Probiotics | 2010 | 42 | $1*10^{10}$ | NCT01225042 | [7] |
| **TNO** | Probiotics | 2011 | 35 | $1*10^{10}$ | NCT01241201 | |
| **PROTETEC** | Probiotics | 2012 | 60 | $3*10^{9}$ | NCT01709266 | [8] |
| **MIRAGE** | Milk phospho-lipids | 2013 | 60 | $1*10^{10}$ | NCT01800396 | [12] |
| **CORAL** | No ingredient intervention | 2016 | 44 | $1*10^{10}$ $5*10^{10}$ | NCT02541695 | To be submitted |
| **PANTER** | Dried bovine colostrum and dried whole egg | 2017 | 72 | $1*10^{10}$ | NCT03301103 | To be submitted |

In the most recent published human intervention study at NIZO food research with this strain performed in 2013,[12] expected adverse events reported the first day after diarrheagenic *E. coli* challenge included abdominal pain (72% of subjects), abdominal distension (52% of subjects), borborygmus (67% of subjects), flatulence (77% of subjects), increased passage of stools (64%

of subjects), loose stools (64% of subjects), nausea (55% of subjects), and urgent defecation (57% of subjects). No serious adverse events were reported during the study. In this study, diarrheagenic *E. coli*-challenge resulted in increased fecal output, lower relative fecal dry weight (at day 1 and 2 after challenge), increased fecal diarrheagenic *E. coli* numbers, and an increase in reported stool frequency and gastro- intestinal complaints at day 1 after challenge. Observed effects of the diarrheagenic *E. coli* challenge were similar to those observed in previous studies.[1, 7, 8]

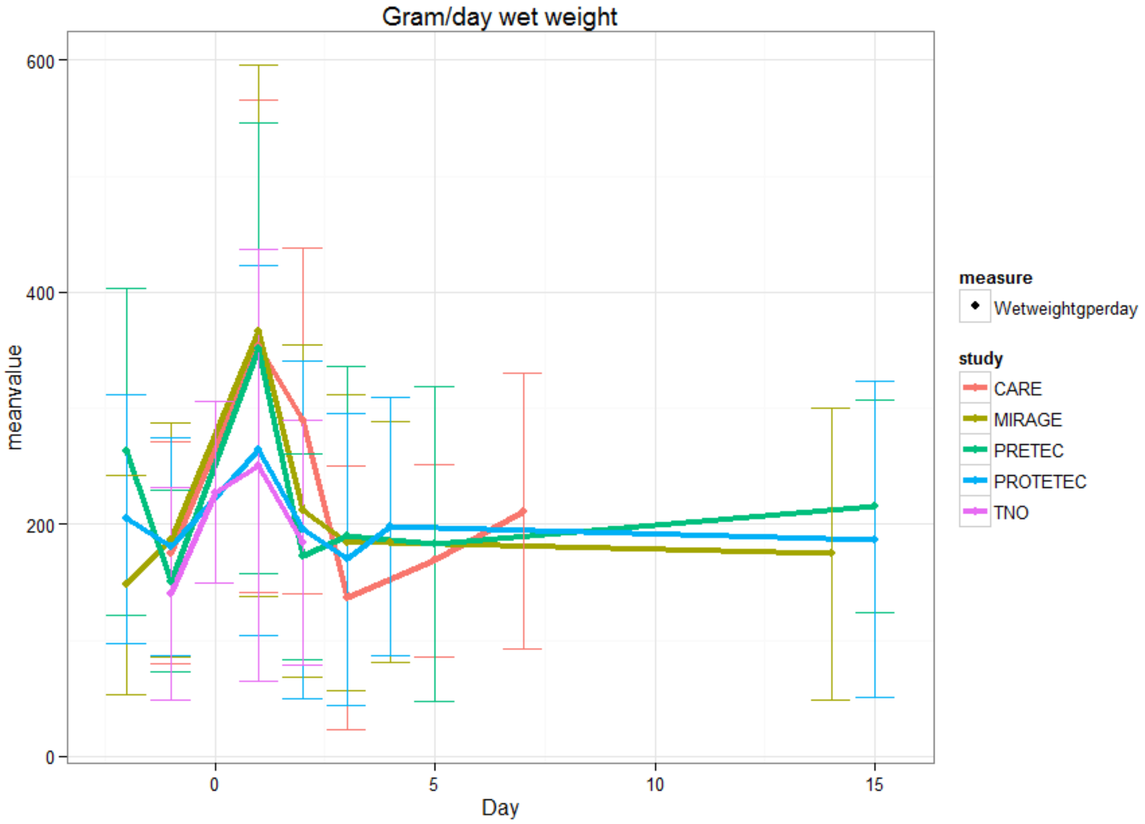


**Effect of diarrheagenic *E. coli* challenge on fecal wet weight (g/day).**
Subjects were orally challenged with $1*10^9$-$1*10^{10}$ colony-forming units (CFU) of diarrheagenic *E. coli* on day 0. Results are expressed as means ± SD.

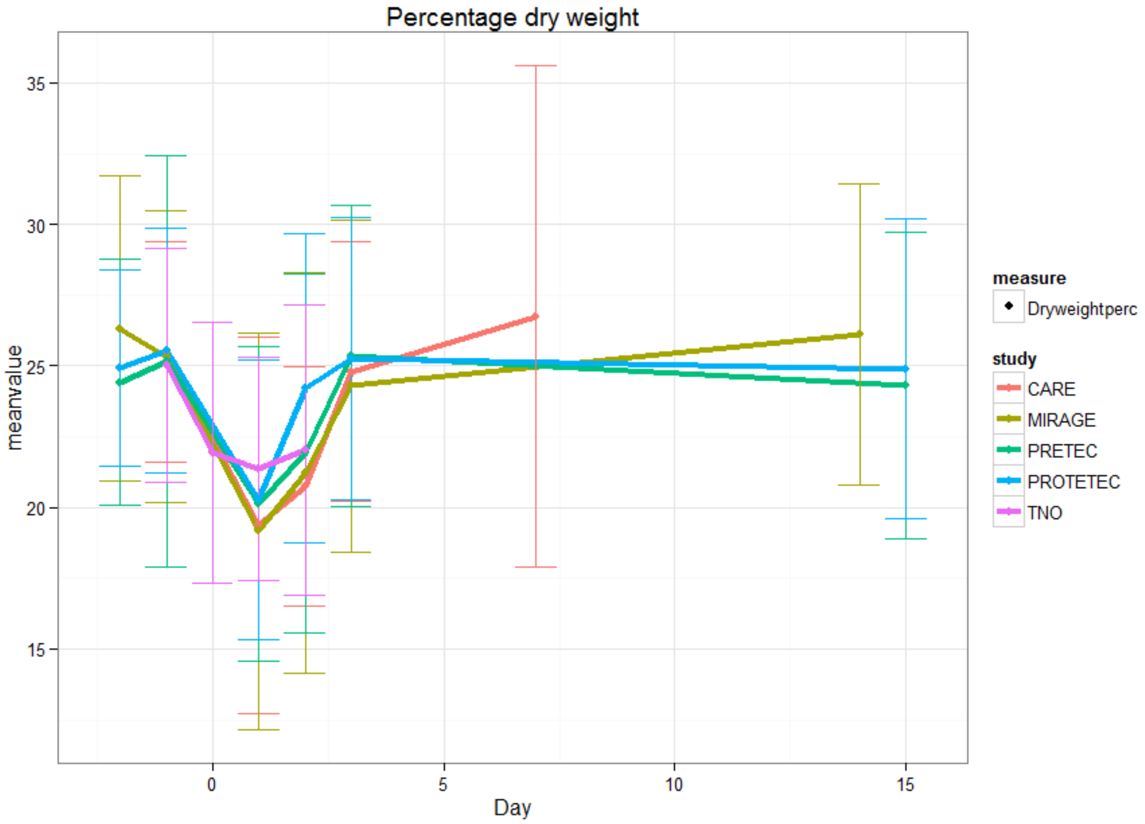


**Effect of diarrheagenic *E. coli* challenge on fecal dry weight (%).**
Subjects were orally challenged with $1*10^9$-$1*10^{10}$ colony-forming units (CFU) of diarrheagenic *E. coli* on day 0. Results are expressed as means ± SD.

### 6.4 Summary of known and potential risks and benefits

The strain used at NIZO food research, the diarrheagenic *E. coli* strain E1392/75-2A, is a spontaneous mutant with deletion of the genes encoding the LT and ST toxins, and can therefore not produce any toxins.

NIZO food research has previously performed 8 intervention studies (n=377 subjects) in humans with *E. coli* strain E1392/75-2A. In the previous 8 intervention studies, the diarrheagenic *E. coli* strain transiently induces symptoms of a food borne infection increasing infectious diarrhea, fecal pathogen excretion, stool frequency, Bristol Stool Score, and reported symptoms. No treatment-related serious adverse events were reported during these studies. All recorded disease episodes were self-limiting and did not require early antibiotic treatment.

*E. coli* strain E1392/75-2A is sensitive to Ciprofloxacin. Ciprofloxacin is commonly used for urinary tract and intestinal infections (traveler's diarrhea) and is a broad-spectrum antibiotic. Because resistance to antibiotics is increasing worldwide, the decision to use an antibiotic should be carefully weighed against the severity of illness and the risk of adverse reactions, such as rash, antibiotic-associated colitis, and vaginal yeast infection [Center for Disease

Control and Prevention]. In the 8 intervention studies performed at NIZO food research all recorded disease episodes were self-limiting and did not require early antibiotic treatment.

As extra safety measure, the *E. coli* inoculate that will be prepared and used for the study will be sent to an external lab on the same day, for analysis of the presence of contaminating pathogens other than *E. coli*. The inoculate needs to be administered on the morning of preparation, and cannot be stored until the results of this analysis will become available. Therefore, the results that will be available after the infection will only guide the medical measures after infection. In case a contaminant is detected, the medical investigator and DSMB will be informed immediately so that they can take medical measures if needed.

There are no direct benefits for the subjects from participation to the MIRRE pilot study other than the following: In previous studies repeated oral administration of diarrheagenic *E. coli* offered 75% protection against challenge with an LT, ST, CFA/II strain. Previous studies with this attenuated strain have shown that a single oral administration leads to a rise of specific serum antibody titers.[1, 7, 8] However, protection would be induced against a very specific (and thus small) group of bacterial pathogens.

### 6.5 Description and justification of route of administration and dosage

At study day 14, after a standardized evening meal and an overnight fast, subjects will receive a single oral dose of the attenuated diarrheagenic *E. coli* strain E1392-75-2A (dose will be either $1*10^{10}$ CFU (n=6), $1*10^{9}$ CFU (n=6), $1*10^{8}$ CFU (n=6), $1*10^{7}$ CFU (n=6), or $1*10^{6}$ CFU (n=6). Oral challenge will occur at 10.00 AM. Under supervision of the project team, subjects will get a $NaHCO_3$ solution (100 mL 2% $NaHCO_3$) to neutralize the gastric acid. After 5 minutes, they get a fruit juice (100 mL) containing the attenuated diarrheagenic *E. coli* strain at the above-mentioned dose. Subjects go home, but are not allowed to drink or eat for 1 hour.
At study day 35, after a standardized evening meal and an overnight fast, all subjects will receive a second inoculation $1*10^{10}$ CFU of the diarrheagenic *E. coli*. E1392/75-2A (all at a dose: $1*10^{10}$ CFU). Oral challenge will occur according to the same procedure as described for day 14.

### 6.6 Dosages, dosage modifications and method of administration

A single oral administration of between $3*10^{9}$ and $5*10^{10}$ CFU of the attenuated diarrheagenic *E. coli* strain E1392-75-2A transiently induces symptoms of a food borne infection increasing infectious diarrhea, fecal pathogen excretion, stool frequency, Bristol Stool Score, reported symptoms, secretory IgA, C-reactive protein, calprotectin and antibody responses.[1, 6, 7] The diarrheagenic *E. coli* strain induces a small increase in average stool frequency from 1 stool/day to 2 stools/day, and an increase in average daily Bristol Stool Score from 4 to 5. Types 1–2 on the Bristol Stool Scale indicate constipation, with 3 and 4 being the ideal stools (as they are easy to defecate while not containing any excess liquid), and 5-7 tending towards diarrhea. All clinical symptoms are transient and last for 1-2 days.

A single oral administration between $3*10^9$ and $5*10^{10}$ CFU of the attenuated diarrheagenic *E. coli* strain has been shown to result in a symptom severity far below those reported in literature for toxin-producing strains.[6, 8, 12] Literature regarding toxin-producing strains indicates that an increase in (extra-)intestinal symptoms can be expected.[6]

For a two times oral administration we know that the current primary dose of infection ($1*10^{10}$ CFU) induces a maximum response, not only in the primary clinical response, but also in the protective secondary response. This results in clear antibody titers that do not further increase upon secondary infection, and absence of clinical symptoms upon a second *E. coli* inoculation (CORAL study).

### 6.7 Preparation and labelling of Investigational Product

The Investigational product is prepared and provided to the subjects as described in the Lab manual Diarrheagenic *E. coli* infection and according to the Standard Operating Procedure SOP P-V003 at NIZO, respectively.

### 6.8 Investigational Product accountability

Dispensing of the Investigational product will be monitored by the Coordinating Investigator and the Investigational product accountability form will be completed:

1. Name & signature of individual dispensing the Investigational product
2. Subject code
3. Date and quantity Dispensed

Destruction of unused Investigational product at the site will be recorded by the Coordinating Investigator.

## 7. NON-INVESTIGATIONAL PRODUCT

Not applicable.

# 8. METHODS

## 8.1 Study parameters/endpoints

### 8.1.1 Main study parameter/endpoint

- Specific antibody titer, serum IgG-CFA/II

### 8.1.2 Secondary study parameters/endpoints

- Relative and total fecal wet weight (Freeze-drying of pooled 24 h fecal samples) at first and second E. coli inoculation
- Stool consistency (Bristol Stool Scale reported by the subjects in the online diary) at first and second E. coli inoculation
- Stool frequency (Stools per day reported by the subjects in the online diary) at first and second E. coli inoculation
- Incidence, duration and severity of Gastro-intestinal symptoms (Gastro-intestinal Symptom Rating Scale reported by the subjects in the online diary) at first and second E. coli inoculation

### 8.1.3 Other study parameters

- Functional immunological assays in peripheral blood mononuclear cells (response to TLR stimulation, phagocytosis, neutrophil function)
- Additional ELISA determinations to identify the response to infection (in plasma)
- Measurement of barrier/inflammation markers in fecal samples

## 8.2 Randomization, blinding and treatment allocation

Subjects will be stratified according to age and BMI (determined at pre-study screening), and subjects will be randomly assigned to one of five treatment groups; $1*10^{10}$ CFU (standard dose); $1*10^{9}$ CFU; $1*10^{8}$ CFU; $1*10^{7}$ CFU; $1*10^{6}$ CFU; n=6 per group.

Stratification and randomization of study subjects, and blinding and labelling of *E. coli* dosages, will be coordinated by a NIZO-project manager not involved in the project. Stratification will be performed manually using the individual data in Excel. Randomization of subjects to treatment group will be performed using a random number generator. Blinding and labeling of the IP will be performed manually by placing a label with the right subject code on the assigned E.coli dosage. The IP is only marked with the subject code.
All researchers of the project team will be kept blind to assignment of treatment, and so will be the study subjects. A printed randomization list, linking the subject to the treatment group, will be stored in a sealed envelope and will be stored in a fire-proof cabinet. A digital version will be stored at a protected location on the local server.

The code of the randomization is kept by the independent project manager and will be broken only if necessary for safety reasons. The investigators and participating subjects will be blinded until the study endpoints have been determined.
The randomization code of the study will be broken after:

- All laboratory reports related to primary and secondary outcomes have been authorized by the Principle Investigator;
- The Data Master File has been documented as meeting the cleaning and approval requirements of the Principle Investigator;
- The finalization and approval of the statistical analysis plan by the Principal Investigator.

## 8.3 Study procedures

### 8.3.1 Information meeting

Information meetings will be organized prior to the study. In these meetings, the investigators will explain the background, objectives and study procedures. When all existing questions of the subjects are answered, subjects willing to participate will be asked to sign both informed consent forms. Subjects will have 1 week to decide on their participation. Subjects will receive one of the signed informed consent forms.

### 8.3.2 Pre-study screening; questionnaire

The subjects will have a pre-study screening before the start of the intervention period. Based on the lifestyle and health questionnaire, a subjects' eligibility will be assessed. If a subject is eligible, the principal investigator will inform him in writing whether he is selected for further screening. If a subject is not eligible, the principal investigator will inform him in writing.

### 8.3.3 Dietary restrictions

*Probiotics and prebiotics/fibers*
Based on published study results and published literature(s), it has been observed that use of probiotics, probiotic based products or prebiotics/fibers during diarrhea episode may affect the duration of the diarrhea. Hence, participating subjects will be instructed to avoid probiotics or probiotic based products and prebiotic/fiber supplements, starting from the run-in period and during the whole study.

*Calcium*
Subjects will be instructed to maintain their habitual diet, except for their dairy intake. In general, Dutch people have a high dairy and thus dietary calcium intake in comparison with many other countries. Dairy has a high calcium content and contributes significantly to total daily calcium intake. From our previous studies, we know that calcium can significantly reduce the gastro-intestinal symptoms induced by the ETEC strain.[1] Calcium is able to reduce luminal cytotoxicity and modulate the intestinal microbiota which subsequently might increase

resistance against gut pathogens. Thus, to ensure the incidence of symptoms similar to previous studies, low-calcium dietary guidelines will be provided from two weeks before study start, to reach a steady state before the infection challenge, and guidelines will be continued during the whole study. These dietary guidelines will limit calcium intake on average to 500 mg/day. This intake is below the recommended daily calcium intake of 1000 mg in The Netherlands, but is quite common in very large population groups around the world (e.g. in Southern Europe, Asian countries, African-Americans, elderly, lactose-intolerant subjects). This temporary restriction in dietary calcium intake is not considered harmful.
Advice will be given on alternatives to replace dairy products.

#### 8.3.4 Online diary and questionnaires

Subjects will report information on stool consistency by using the Bristol stool scale,[13] stool frequency, and registration of medication intake in an online subject diary. Moreover, in the online diary, subjects will record frequency and severity of symptoms related to Reflux, Abdominal pain, Indigestion, Diarrhea and Constipation by the validated Gastro-intestinal Symptom Rating Scale (GSRS).[14] The GSRS is a disease-specific instrument of 15 items combined into five symptom clusters. The GSRS has a seven-point graded Likert-type scale where 1 represents absence of troublesome symptoms and 7 represents very troublesome symptoms.
The following software will be used to record this information: De research manager, http://deresearchmanager.nl, Deventer, The Netherlands; NEN 7510 and ISO 27001 compliant. Cloud9 is ISO 27001 certified.
Compliance of the subjects to reporting these parameters, through the online diary and questionnaires, will be monitored daily by the Study Coordinator. In case information has been inadequately captured, the Study Coordinator will follow up by e-mail or phone to verify the entry.

#### 8.3.5 Diarrheagenic *E. coli* inoculation

At study day 14, after an overnight fast, subjects will receive a single oral dose of the attenuated diarrheagenic *E. coli* strain E1392/75-2A (5 dosages: $1*10^{10}$ / $1*10^{9}$ / $1*10^{8}$ / $1*10^{7}$ / $1*10^{6}$ CFU). Oral challenge will occur in the morning. Under supervision of the project team, subjects will get a NaHCO3 solution (100 mL 2% NaHCO3) to neutralize the gastric acid. After 5 minutes, they get a fruit juice (100 mL) containing the attenuated diarrheagenic E. coli strain at the above-mentioned dosages. Subjects go home, but are not allowed to drink or eat for 1 hour following diarrheagenic *E. coli* inoculation.
At study day 35, after an overnight fast, subjects will receive a second single oral dose of the attenuated diarrheagenic *E. coli* strain E1392/75-2A (all at a dose: $1*10^{10}$ CFU). Oral challenge will occur according to the same procedure as described for day 14.

#### 8.3.6 Biological sample collection

Blood samples will be taken by qualified staff at 6 timepoints during the study (40 mL on day 14, 28, 35, 49; 20 mL on day 21, 42; see study schedule under paragraph 3).
On 1 day before (day 12 or day 13; day 33 or 34) and 3 days after each inoculation day (day 15-17; day 36-38; 8 days in total), 24 h fecal samples will be collected. All materials and information needed for proper collection of the fecal samples will be supplied by NIZO and delivered to the subjects. Feces will be frozen at subjects home immediately after defecation. Subjects will be asked to store feces in mini-freezers, supplied by NIZO. Every 2-3 days, the frozen feces will be transported to the lab, weighed, and homogenized. Homogenized fecal sub-samples will be frozen and stored at -20°C for later analyses.

Method to collect a complete stool is described below:
All materials to collect all separate daily stools will be provided to the subjects. Subjects will need to collect all 24h stool samples separately using the Fecotainer (picture left below). Subjects will need to cover the Fecotainer with a collection bag. After collection, the bag will be closed tightly using a tie-wrap (picture right below) and an additional bag will be used to cover the sample. Thereafter, the stool sample needs to be frozen in the subject's mobile mini freezer as provided by NIZO. The 24h fecal samples will be transported to NIZO on dry ice by a courier service.

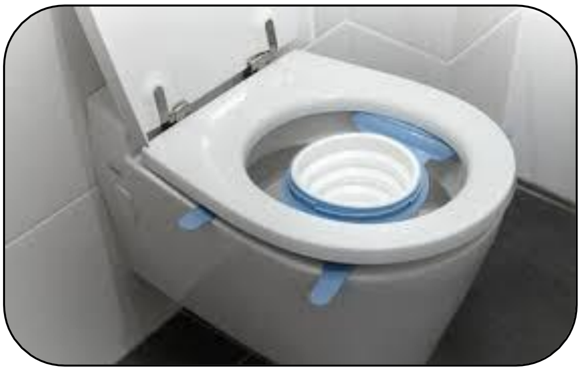

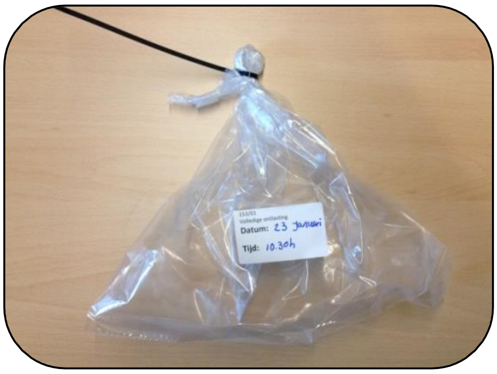


### 8.4 Withdrawal of individual subjects

Subjects can leave the study at any time for any reason if they wish to do so without any consequences. The medical investigator can decide to withdraw a subject from the study for urgent medical reasons, in consultation with the principal investigator. The principal investigator can decide to withdraw a subject from the study if subject does not comply with the rules and regulations of the study.

## 8.5 Replacement of individual subjects after withdrawal

If subjects withdraw before start of the dietary guidelines (two weeks before the first *E. coli* inoculation, subjects will be replaced. Subjects not completing the study for any reason will be considered as drop-outs and will not be replaced.

## 8.6 Follow-up of subjects withdrawn from treatment

After possible withdrawal, no follow-up of participants will take place, except in cases of withdrawal for medical reasons.

## 8.7 Premature termination of the study

The study will only be terminated prematurely if an acute SAE is reported on day 14, 15 or 16 after diarrheagenic *E. coli* infection, which, to the opinion of the medical investigator or the DSMB, might be related to the *E. coli* infection. As after secondary infection no further interventions will be done except for biological sampling, an acute SAE related to the *E. coli* infection after the secondary infection will only result in premature termination of the study for that subject, whereas other subjects will continue. Although considered unlikely, in case of premature termination of the study by the investigators, all subjects will be informed and will be paid pro rata for the part of the study that has already been performed.

# 9. SAFETY REPORTING

## 9.1 Temporary halt for reasons of subject safety

In accordance to section 10, subsection 4, of the WMO, the sponsor will suspend the study if there is sufficient ground that continuation of the study will jeopardise subject health or safety. The sponsor will notify the accredited METC without undue delay of a temporary halt including the reason for such an action. The study will be suspended pending a further positive decision by the accredited METC. The investigator will take care that all subjects are kept informed.

## 9.2 AEs, SAEs and SUSARs

### 9.2.1 Adverse events (AEs)

Adverse events are defined as any undesirable experience occurring to a subject during the study, whether or not considered related to the investigational product (*E. coli* infection). All adverse events reported spontaneously by the subject or observed by the investigator or his staff will be recorded.

### 9.2.2 Serious adverse events (SAEs)

A serious adverse event is any untoward medical occurrence or effect that
results in death;

- is life threatening (at the time of the event);
- requires hospitalisation or prolongation of existing inpatients' hospitalisation;
- results in persistent or significant disability or incapacity;
- is a congenital anomaly or birth defect; or
- any other important medical event that did not result in any of the outcomes listed above due to medical or surgical intervention but could have been based upon appropriate judgement by the investigator.

An elective hospital admission will not be considered as a serious adverse event.
All serious adverse events will be reported by the Principal Investigator to the Sponsor without undue delay after obtaining knowledge of the events.

The Principle Investigator will report the SAEs through the web portal ToetsingOnline to the accredited METC that approved the protocol, within 7 days of first knowledge for SAEs that result in death or are life threatening followed by a period of maximum of 8 days to complete the initial preliminary report. All other SAEs will be reported within a period of maximum 15 days after the sponsor has first knowledge of the serious adverse events.

All (S)AEs will be recorded (as reported by the subjects by phone or as reported online by the subjects), using the following software: De research manager, http://deresearchmanager.nl, Deventer, The Netherlands; NEN 7510 and ISO 27001 compliant. Cloud9 is ISO 27001 certified. AEs are graded as follows:

- Mild: sign or symptom, usually transient, requiring no special treatment and generally not interfering with usual activities.
- Moderate: sign or symptom, which may be ameliorated by simple therapeutic measures, may interfere with usual activity.
- Severe: sign or symptom intense or debilitating and interfering with usual activities without being immediately life-threatening. Recovery is usually aided by therapeutic measures.
- Very severe: sign or symptom life-threatening. Urgent intervention indicated.

#### 9.2.3 Suspected unexpected serious adverse reactions (SUSARs)

Not applicable

### 9.3 Annual safety report

Not applicable

### 9.4 Follow-up of adverse events

All AEs will be followed until they have abated, or until a stable situation has been reached. Depending on the event, follow up may require additional tests or medical procedures as indicated, and/or referral to the general physician or a medical specialist.
SAEs need to be reported till end of study within the Netherlands, as defined in the protocol

### 9.5 Data Safety Monitoring Board (DSMB)

A DSMB will be established for this study to perform ongoing safety surveillance and to perform interim analyses on the safety data. The DSMB charter has been included in the dossier (see Chapter K of the IRB dossier). The DSMB is an independent committee completely unblinded for treatment allocation and is composed of the following persons:

1.(pending review and approval of the DSMB charter)
Ineke Klöpping, MD
Research Physician
Division of Human Nutrition
Wageningen University
PO Box 17
6700 AA Wageningen

2. (pending review and approval of the DSMB charter)
Meta Roestenberg, MD
Leids Universitair Medisch Centrum
Department of Infectious Diseases
Albinusdreef 2
2333 ZA Leiden

The interim analysis will be performed two days after each diarrheagenic *E. coli* challenge. The DSMB members will assess safety in a unblinded manner.
The interim analysis will focus on the following issues: adverse events: type, severity, duration, action taken and attributability to the diarrheagenic *E. coli* challenge.

The advice(s) of the DSMB will only be sent to the Principle Investigator of the study. Should the Principle Investigator decide not to fully implement the advice of the DSMB, the Principle Investigator will send the advice to the reviewing METC, including a note to substantiate why (part of) the advice of the DSMB will not be followed.

## 10. STATISTICAL ANALYSIS

Since the present study concerns a pilot study, obtaining statistical significance is not specifically required. Obviously, statistical analysis will be performed to analyze the power of the outcome and to power a potentially subsequent study.
Intention-to-Treat (ITT) analysis will be performed for all aims defined. To avoid a loss in sensitivity, no imputation will be done to minimize regression to the mean.

**Normality**

Normality in the data will be checked for all study parameters via Shapiro-Wilk normality test and outlier presence by Grubbs test. The type of statistical analysis will be adapted according to the distribution characteristics in the data.

**Baseline parameters**

For the descriptive statistics, values will be given as mean ± standard deviation or median and interquartile ranges, depending on their distribution characteristics. Differences in these descriptive values at the start will be analyzed via Mann-Whitney U-test or unpaired t-test, depending on the respective distribution characteristics.
Descriptive information will be showing: mean, standard deviation (SD), standard error of the mean (SEM), median, minimum and maximum, number of non-missing and missing observations.

**Missing data**

If data are missing, statistical analyses will be performed without these data, and number of missing values will be reported.
If in total more than 50% of the data for a particular subject is missing, the respective subject will not be included in the ITT analysis. That decision will be taken before unblinding the data and before starting the statistical analyses.

**Statistical program**

Study results will be statistically analyzed applying STATA (version 12, StataCorp, College Station, TX, USA) or a comparable software program, and GraphPad (version 6, GraphPad Prism, LaJolla, CA, USA). Data handling will be addressed in Excel (Microsoft, Redmond, Washington, US) and checked within STATA, version 12 or a comparable software program.

**Study parameters**

The main study parameter is:

- Specific antibody titer, serum IgG-CFA/II at the secondary infection.

Secondary study parameters are:

- Relative and total fecal wet weight (Freeze-drying of pooled 24 h fecal samples) at first and second E. coli inoculation

- Stool consistency (Bristol Stool Scale reported by the subjects in the online diary) at first and second E. coli inoculation
- Stool frequency (Stools per day reported by the subjects in the online diary) at first and second E. coli inoculation
- Incidence, duration and severity of Gastro-intestinal symptoms (Gastro-intestinal Symptom Rating Scale reported by the subjects in the online diary) at first and second E. coli inoculation

Tertiary explorative study parameters are:

- Functional immunological assays in peripheral blood mononuclear cells (response to TLR stimulation, phagocytosis, neutrophil function)
- Additional ELISA determinations to identify the response to infection (in plasma)
- Measurement of barrier/inflammation markers in fecal samples

## 10.1 Primary study parameter(s)

Investigating the effect of the primary inoculation dose on the specific antibody titer, serum IgG-CFA/II, at the secondary *E. coli* infection:

- Titers will be 10log-transformed. Delta of 10log-transformed titers will be calculated at day 35 versus day 14. The delta-values of the five different E. coli dosage groups will be analyzed using General Estimating Equations (GEE analysis) in the Gaussian mode, since it is believed that the transformation yields normality in the dependent parameter, followed by Dunnett's multiple comparison test to evaluate a dose to dose comparison. In the GEE analysis next to dose and time as independent parameters various potentially confounding parameters will be integrated, as there are: age, BMI, starting value of serum titer at day 14, etc. The GEE will be performed in a repeated measures, stepwise multifactorial approach.
- To analyze the kinetics of the antibody response over time, the delta of 10log-transformed titers will be calculated at days 21, 28, 35, 42 and 49 versus day 14. The outcome in delta values in the five different E. coli dosage groups will be analyzed using stepwise, repeated measures GEE, with time and dose as independent parameters combined with confounding factors, followed by Dunnett's multiple comparison test to analyze a dose by dose comparison.

## 10.2 Secondary study parameters

As the variation in the secondary (clinical) parameters is expected to be high, the secondary study parameters will only be analyzed exploratively:

- The effect of the dose of primary E. coli infection on relative and total fecal wet weight on day 15-17 and day 36-38 (delta or fold-change post- versus pre-challenge), applying either Gaussian or non-Gaussian distribution dependent on the nature of distribution in the dependent parameter

- The effect of the dose of primary E. coli infection on Stool consistency (Bristol Stool Scale reported by the subjects in the online diary) on day 14-17 and day 35-38 (delta or fold-change post- versus pre-challenge), applying Poisson distribution due to the discrete nature in the data of the dependent parameter
- The effect of the dose of primary E. coli infection on stool frequency (stools per day reported by the subjects in the online diary) on day 14-17 and day 35-38 (delta or fold-change post- versus pre-challenge), applying Poisson distribution due to the discrete nature in the data of the dependent parameter
- The effect of the dose of primary E. coli infection on incidence, duration and severity of Gastro-intestinal symptoms (Gastro-intestinal Symptom Rating Scale reported by the subjects in the online diary) on day 14-17 and day 35-38 (delta or fold-change post- versus pre-challenge), applying Gaussian or non-Gaussian distribution depending on the nature in the distribution of the data of the dependent outcome
- Correlation of the clinical symptoms as measured by fecal wet weight, stool consistency, stool frequency and GSRS, with the kinetics and levels of antibody responses (primary outcome) over time.

Analysis will be performed via stepwise, repeated measures GEE applying time and dose as independent parameters, combined with the outcome in potentially confounding factors.

As a clear direction of effect is expected (less protection at secondary infection with decreasing dose of primary E. coli infection), statistical tests will be conducted one-sided. A p-value less than 0.05 will be considered as statistically significant.

Detailed descriptions of statistical analysis methods and data conventions will be described later in a Statistical Analysis Plan (SAP).

### 10.3 Other study parameters

The pilot study will have an additional exploratory character. For explorative purposes, at 4 timepoints (day 14, 28, 35, 49), 20 mL extra of blood will be drawn (40 mL instead of 20 mL). If the primary and secondary outcome parameters show a clear dose-dependent effect in clinical symptoms between the different dosage groups, or if unexpected interesting effects are observed that warrant further investigation, additional biomarkers that may be associated with the strength of the immune response will be explored. This may include additional serum/plasma markers of infection (e.g. IgA antibodies, inflammatory cytokines/chemokines/proteins), and fecal markers of infection and barrier function (e.g. calprotectin, zonulin, α1-antitrypsin). If logistically feasible (resources should be available on the study days as it requires freshly collected blood), markers for immune function (e.g. TLR responsiveness, phagocytosis, neutrophil function) will also be analyzed. For these tertiary parameters, we will isolate PBMCs and store serum/plasma and fecal material.

## 10.4 Interim analysis

Not applicable.

# 11. ETHICAL CONSIDERATIONS

## 11.1 Regulation statement

Subjects will only participate to the MIRRE pilot study upon providing written informed consent. The study will be conducted according to the principles of the Declaration of Helsinki (Fortaleza, Brazil, October 2013) and according to the Dutch Medical Research involving Human Subjects Act.

## 11.2 Recruitment and consent

Candidates will be recruited by Link2Trials B.V. (Haarlem), with support of NIZO, from the Wageningen/Ede environment. Subjects will be recruited by advertisements in regional newspapers and on social media, and, if needed, by advertisements in local newspapers and by posters mounted in public buildings. Candidate subjects will receive written information of the study (see Chapter E IRB dossier). Texts for the various recruitment materials (advertisements, letters, website etc) are supplied as part of the dossier in Chapter E.

Link2Trials B.V. will use a pre-selection questionnaire based on a selection of major in- and exclusion criteria, in order to restrict the number of unnecessary screenings. It is expected that, based on general information and main selection criteria, about 140 candidates will be invited to attend an oral information session at NIZO (Ede). During these sessions, the candidates will be informed verbally on the aim, study procedures, the constraints and insurance of the study, and receive a hardcopy of the information for subjects. Candidates will have the opportunity to ask questions to research team members or consult the independent physician mr. D. Lansdorp (see Chapter E IRB dossier).
When all existing questions of the subjects are answered, subjects willing to participate will be asked to sign two copies of the informed consent forms. Subjects will have 1 week to decide on their participation. Subjects will receive one of the signed informed consent forms. After signing the informed consent, subjects will be asked to complete a health and lifestyle questionnaire with more specific questions related to in- and exclusion criteria at the oral information session at NIZO (Ede).
The subjects will have a pre-study screening before the start of the intervention period. Based on the lifestyle and health questionnaire, subject's eligibility will be assessed. If a subject is eligible, the principal investigator will inform him in writing whether he/she is invited to participate or is not selected. If a subject is not eligible, the principal investigator will inform him in writing.

Subjects that fulfill all eligibility criteria based on the lifestyle and health questionnaire will be included in the study.
The principal investigator will inform in writing the general practitioner of each subject. who has signed the informed consent form and is selected to participate in the study, on study

participation. Any new significant findings reported in scientific literature that might affect the subject's participation in the study will be communicated to the subject.

## 11.3 Objection by minors or incapacitated subjects

Not applicable.

## 11.4 Benefits and risks assessment, group relatedness

*Benefits*

There are no direct benefits for the subjects from participation to the MIRRE pilot study other than the following: In previous studies repeated oral administration of diarrheagenic E. coli offered 75% protection against challenge with an LT, ST, CFA/II strain.[5] Previous studies with this attenuated strain have shown that a single oral administration leads to a rise of specific serum antibody titers.[1, 7, 8] However, protection would be induced against a very specific (and thus small) group of bacterial pathogens.

*Risks*

Over the past 40 years, the enterotoxigenic E. coli (ETEC) human challenge model has been used to elucidate the pathogenesis and immune responses associated with ETEC infection as well as to test the efficacy of investigational drugs and vaccines. A systematic review of the published and unpublished literature to evaluate specific outcomes in subjects participating in experimental ETEC infection studies using the accepted principles of good methodological design was published previously by Porter et al (2011).[6]

Unlike the strains used in this systematic review, the strain used at NIZO food research, is a spontaneous mutant unable to produce toxins. The basic concept of the diarrheagenic *E. coli* strain challenge study we have developed at NIZO food research is that we have selected a well-characterized, antibiotic susceptible organism that has been associated with very mild diarrhea and gastrointestinal symptoms (severity and duration).[1, 7, 8]

NIZO food research has previously performed 8 nutritional intervention studies (n=377 subjects) in humans with *E. coli* strain E1392/75-2A. In the previous studies, all recorded disease episodes were self-limiting and did not require early antibiotic treatment. No treatment-related serious adverse events were reported during these studies. The diarrheagenic *E. coli* strain E1392/75-2A strain is sensitive to Ciprofloxacin which is a commonly used antibiotic in case of treatment of this kind of *E. coli* infections.

As extra safety measure, the *E. coli* inoculate that will be prepared and used for the study will be sent to an external lab on the same day, for analysis of the presence of contaminating pathogens other than *E. coli*. The inoculate needs to be administered on the morning of preparation, and cannot be stored until the results of this analysis will become available. Therefore, the results that will be available after the infection will only guide the medical measures after infection. In case a contaminant is detected, the medical investigator and DSMB will be informed immediately so that they can take medical measures if needed.

The amount of blood sampled during the study is 2 x 20 mL and 4 x 40 mL per sampling timepoint (200 mL in total). This is far less than the amount of blood collected for blood donation purposes, at a single occasion (500 mL). Blood sampling can lead to hematoma, or to dizziness.
Based on these considerations, to our opinion, the risks for participation in this study are low, and we have made every effort to minimize potential risks. Therefore, we feel that the remaining risks are acceptable and do not outweigh the scientific relevance of this study.

### 11.5 Compensation for injury

NIZO food research has a liability insurance which is in accordance with article 7 of the WMO. NIZO food research also has an insurance which is in accordance with the legal requirements in The Netherlands (Article 7 WMO and the Measure regarding Compulsory Insurance for Clinical Research in Humans of 14 November 2014). This insurance (HDI Gerling, Amsterdam, The Netherlands) provides cover for damage to research subjects through injury or death caused by the study or the pre-study screening.

This insurance provides cover for damage to research subjects through injury or death caused by the study.

1. € 650.000,-- (i.e. six hundred and fifty thousand Euro) for death or injury for each subject who participates in the Research;
2. € 5.000.000,-- (i.e. five million Euro) for death or injury for all subjects who participate in the Research;
3. € 7.500.000,-- (i.e. seven million five hundred thousand Euro) for the total damage incurred by the organization for all damage disclosed by scientific research for the Sponsor as ‘verrichter’ in the meaning of said Act in each year of insurance coverage.
4. € 7.500.000,-- (i.e. seven million five hundred thousand Euro) for the total damage that becomes apparent after the insurance period.

The insurance applies to the damage that becomes apparent during the study or within 4 years after the end of the study.

### 11.6 Incentives (if applicable)

Time investment:
The main burden of this study consists of the time involved in participation. This will be in total 15 hours. Details of the time investments are listed below:

Detailed time investment for each participant

| Activity | Frequency | Minutes | Total minutes |
|---|---|---|---|
| Information meeting | 1 | 120 | 120 |
| Screening (questionnaire) | 1 | 30 | 30 |

| | | | |
|---|---|---|---|
| Study Stool collection kit and meal delivery | 2 | 30 | 60 |
| Study Questionnaires | 14 | 30 | 420 |
| Study Fecal sample collection | 14 | 15 | 210 |
| Study dietary guidelines | 49 | 10 | 490 |
| Study *E. coli* inoculation | 2 | 120 | 240 |
| Study blood sampling visit | 6 | 15 | 90 |
| Total minutes | | | 1660 minutes (~28 hours) |

Subjects will receive €700.= after full completion of the study, plus traveling expenditures (€0.19 per km), which will be subject to taxes. If a subjects ends the participation earlier, they will receive only part of the financial reward. The reward is intended to compensate the subjects for their time spent on study activities, e.g. filling in the online diaries and the collection of biological samples. Subjects will be informed about payment criteria orally and in writing.

Subjects attending the oral information session will receive €10,= in addition.

Participants are free to withdraw from further participation for any reason and at any time during the trial. Subjects who drop out during the study will be paid a sum in accordance with study participation:

1. Full amount (€700,= plus travel costs), if participation is ended by:
   a. The medical investigator on medical grounds
   b. The principal investigator, based on deviations in the study conduct (to be judged by the project leader)
   c. Premature discontinuation by sponsor and/or NIZO.
   d. Circumstances beyond one's control.
2. €10,=, if:
   a. Subject is attending the oral information session.
3. No payment if:
   a. The Principal Investigator prematurely ends the subject's participation on grounds of consistently not complying with the rules and regulations of the study or misconduct.
4. Pro rata sum:
   a. If withdrawing on own initiative before the end of the study, participants will receive a proportional payment for the effort they made (€2.= for each study day, €100.= for each E. coli inoculation day, and €15.= for each blood sampling visit).

# 12. ADMINISTRATIVE ASPECTS, MONITORING AND PUBLICATION

## 12.1 Handling and storage of data and documents

All data will be handled confidentially and in compliance with the Dutch Personal Data Protection Act. A subject identification code list will be used to link the data to the subject. The code is not based on the patient initials and birth-date. The key to the code will be safeguarded by the principal investigator and Coordinating Investigator.

After signing the informed consent form, subjects will be allocated to a pre-entry number consisting of the study code (Study number, followed by a slash ("/"), followed by S and a 3-digit number starting at S501. After inclusion of all subjects, subjects will be allocated to an entry number. Entry numbers will consist of the following study code: Study number, followed by a slash ("/"), followed by a 2-digit number (for example 183/01).

Biological samples will be stored for 10 years after the study has ended at NIZO. Biological samples obtained will be stored under appropriate conditions to enable ad hoc retrospective research in the future, e.g. for new biomarker validation. In all study reports, only group averages or anonymous data will be presented. The principal investigator, study coordinator and the lab scientific assistants are the only members of the study team having access to the biological samples. Samples are only marked with the subject code.

Subjects will be asked to fill in their diaries online. To this end, subjects will receive a unique username and password. The subjects will only have access to their own diary. The principal investigator, study coordinator and data manager are the only members of the study team having access to the electronic data base. Data are only marked with the subject code. The following software will be used: De research manager, http://deresearchmanager.nl, Deventer, The Netherlands; NEN 7510 and ISO 27001 compliant. Cloud9 is ISO 27001 certified.

## 12.2 Monitoring and Quality Assurance

Quality control of the study will be done by a qualified internal employee at NIZO, not belonging to the study team. Activities will be described in the project management plan, and can include the following: Investigator and Site Responsibilities, Protocol Review, Informed Consent Process, Study Documentation, Electronic Case Report Form, Safety Reporting, Source Documentation, Laboratory Procedures.

## 12.3 Amendments

A 'substantial amendment' is defined as an amendment to the terms of the METC application, or to the protocol or any other supporting documentation, that is likely to affect to a significant degree:

- the safety or physical or mental integrity of the subjects of the trial;
- the scientific value of the trial;

- the conduct or management of the trial; or
- the quality or safety of any intervention used in the trial.

All substantial amendments will be notified to the METC and to the competent authority.

Non-substantial amendments will not be notified to the accredited METC and the competent authority, but will be recorded and filed by the sponsor.

### 12.4 Annual progress report

Not applicable.

### 12.5 Temporary halt and (prematurely) end of study report

The investigator/sponsor will notify the accredited METC of the end of the study within a period of 8 weeks. The end of the study is defined as the last subject's last visit.
The sponsor will notify the METC immediately of a temporary halt of the study, including the reason of such an action.
In case the study is ended prematurely, the sponsor will notify the accredited METC within 15 days, including the reasons for the premature termination.
Within one year after the end of the study, the investigator/sponsor will submit a final study report with the results of the study, including any publications/abstracts of the study, to the accredited METC.

### 12.6 Public disclosure and publication policy

The study protocol will be registered at a public database (www.clinicaltrials.gov) after approval from the accredited METC is received.
NIZO will not unreasonably withhold the public disclosure of study results. Public disclosure may include:

- Registration of the study in a clinical trial register (clinicaltrials.gov);
- Summarized anonymous study results issued on a NIZO website, or an international results website (clinicaltrials.org);
- Oral or poster presentation at conferences, symposia or other public meetings;
- Full publication in peer-reviewed scientific journals.

Anonymized results of the study will be publicly disclosed by NIZO within 1 year after the final report has been issued.

# 13. STRUCTURED RISK ANALYSIS

## 13.1 Potential issues of concern

*a. Level of knowledge about mechanism of action*

In the classical paradigm of ETEC pathogenesis these organisms were thought to adhere to the small intestine via fimbrial colonization factors where they release their enterotoxins through cell lysis to produce disease. Over time, however, a more complex picture has emerged with the identification of specific secretion systems to export toxins, as well as other virulence factors that facilitate the delivery of these toxins including novel adhesins. Other molecules, including the extracellular protease EatA10 which appears to modulate adhesion and intestinal colonization by ETEC have also been demonstrated to play a role. Furthermore, a number of chromosomal factors are thought to be involved in virulence, e.g., the invasin Tia; the TibA adhesin/invasin; and LeoA, a GTPase with unknown function.

The chromosome of E. coli H10407 is most closely related to those of nonpathogenic E. coli strains. The factors mediating diarrhea are not chromosomally encoded, indicating that the essential virulence factors are encoded on the plasmids. Collectively, the pathogenesis of these organisms appears to be considerably more complex than previously believed.[15]

*b. Previous exposure of human beings with the test product(s) and/or products with a similar biological mechanism*

- ETEC challenge models at other research groups

Over the past 40 years, the enterotoxigenic *E. coli* (ETEC) human challenge model has been used to elucidate the pathogenesis and immune responses associated with ETEC infection as well as to test the efficacy of investigational drugs and vaccines. A systematic review of the published and unpublished literature to evaluate specific outcomes in subjects participating in experimental ETEC infection studies using the accepted principles of good methodological design was published previously by Porter et al (2011).[6]

- Attenuated diarrheagenic E. coli challenge model

Unlike the strains used in this systematic review, the strain used at NIZO is a spontaneous mutant that is unable to produce toxins. The strain has a spontaneous deletion of the genes encoding the LT and ST toxins.However, it continues to express CFA/II and provides 75% protection against challenge with an LT, ST, CFA/II strain.[5]

The basic concept of the diarrheagenic *E. coli* strain challenge study developed at NIZO is based on the selection of a well-characterized, antibiotic susceptible organism that has been associated with very mild diarrhea and gastrointestinal symptoms (severity and duration).[1, 7, 8]

NIZO has previously performed 8 nutritional intervention studies in humans with this *E. coli* strain E1392/75-2A. All recorded disease episodes were self-limiting and did not require early antibiotic treatment. The diarrheagenic *E. coli* strain transiently (for 1-2 days) induces symptoms of a food borne infection. It increases infectious diarrhea, stool frequency, and decreases stool consistency.[1, 6, 7] The diarrheagenic *E. coli* strain induces a small increase in

average stool frequency from 1 stool/day to 2 stools/day, and an increase in average daily Bristol Stool Score from 4 to 5. Types 1–2 on the Bristol Stool Scale indicate constipation, with 3 and 4 being the ideal stools (as they are easy to defecate while not containing any excess liquid), and 5-7 tending towards diarrhea.

*c. Can the primary or secondary mechanism be induced in animals and/or in ex-vivo human cell material?*
Although the diarrheagenic E. coli strain showed qualitatively similar effects with supplemental calcium phosphate in rats compared to in humans,[1] verifying the observed effects in humans is a requirement for supporting a subsequent health claim.

*d. Selectivity of the mechanism to target tissue in animals and/or human beings*
Not applicable

*e. Analysis of potential effect*
NIZO has previously performed 8 nutritional intervention studies in humans with this E. coli strain E1392/75-2A. All recorded disease episodes were self-limiting and did not require early antibiotic treatment. We expect that a single oral administration of $1*10^{10}$ CFU of the attenuated diarrheagenic *E. coli* strain will result in a symptom severity far below those reported in literature for toxin-producing strains.[6]

*f. Pharmacokinetic considerations*
Not applicable

*g. Study population*
30 healthy human subjects recruited from the Wageningen/Ede (The Netherlands) area who fulfill all of the inclusion criteria and none of the exclusion criteria.

*h. Interaction with other products*
Not applicable

*i. Predictability of effect*
NIZO has previously performed 8 nutritional intervention studies in humans with this *E. coli* strain E1392/75-2A. Results obtained in these studies show that the effect of the diarrheagenic *E. coli* strain is reproducible between these studies. The diarrheagenic *E. coli* strain transiently induces symptoms of a food borne infection increasing infectious diarrhea, fecal pathogen excretion, stool frequency, Bristol Stool Score, reported symptoms, secretory IgA, C-reactive protein, calprotectin and antibody responses.[1, 6, 7] The diarrheagenic *E. coli* strain induces a small increase in average stool frequency from 1 stool/day to 2 stools/day, and an increase in average daily Bristol Stool Score from 4 to 5. Types 1–2 on the Bristol Stool Scale indicate constipation, with 3 and 4 being the ideal stools (as they are easy to defecate

while not containing any excess liquid), and 5-7 tending towards diarrhea. All clinical symptoms are transient and last for 1-2 days.

*j. Can effects be managed?*
In previous studies, all recorded disease episodes after diarrheagenic *E. coli* challenge were self-limiting and did not require early antibiotic treatment. E. coli strain E1392/75-2A is sensitive to Ciprofloxacin. Ciprofloxacin is commonly used for urinary tract and intestinal infections (traveler's diarrhea) and is a broad-spectrum antibiotic. Because resistance to antibiotics is increasing worldwide, the decision to use an antibiotic should be carefully weighed against the severity of illness and the risk of adverse reactions, such as rash, antibiotic-associated colitis, and vaginal yeast infection [Center for Disease Control and Prevention].

## 13.2 Synthesis

The main issue of concern of the approach in the current study is the incidence of symptoms after *E. coli* infection, which is relatively high. The percentages of subjects affected in the recent studies was: abdominal pain (~70-75% of subjects), abdominal distension (~50-55% of subjects), borborygmus (~65-70% of subjects), flatulence (~75-80% of subjects), increased passage of stools (~60-65% of subjects), loose stools (~60-65% of subjects), nausea (~55-60% of subjects), and urgent defecation (~55-60% of subjects). However, reported frequency and severity of clinical symptoms throughout all studies was low as compared to studies using wild-type strains, and duration of these symptoms is short (on average 2 days), and did not require early antibiotic treatment.
In the current pilot study, two subsequent infections will be provided. The primary infection dose will be different in the 5 groups (from $1*10^6$ – $1*10^{10}$ CFU), which is expected to result in a dose-dependent decrease of symptoms with decreasing dose, with the maximum of symptoms similar to previous studies. The secondary infection will be given at the standard dose of $1*10^{10}$ CFU. It is expected that the subjects that received the lowest dose at primary infection will have more symptoms at the secondary infection, whereas the subjects that received the highest dose at primary infection will hardly have any symptoms at secondary infection. It is expected that all symptoms at secondary infection will be similar to or lower than those in previous studies.
Based on the above-given information, the risks for participation in this study are estimated to be small, and we have made every effort to minimize potential risks. Therefore, we feel that the remaining risks are acceptable and do not outweigh the scientific relevance of this study.

# 14. REFERENCES

1. Bovee-Oudenhoven IM, Lettink-Wissink ML, Van Doesburg W, Witteman BJ, Van Der Meer R. Diarrhea caused by enterotoxigenic Escherichia coli infection of humans is inhibited by dietary calcium. Gastroenterology 2003;125:469-76.
2. Doorduyn Y, Van Pelt W, Havelaar AH. The burden of infectious intestinal disease (IID) in the community: a survey of self-reported IID in The Netherlands. Epidemiol Infect 2012;140:1185-92.
3. Kollaritsch H, Paulke-Korinek M, Wiedermann U. Traveler's Diarrhea. Infect Dis Clin North Am 2012;26:691-706.
4. Rendi-Wagner P, Kollaritsch H. Drug prophylaxis for travelers' diarrhea. Clin Infect Dis 2002;34:628-33.
5. Tacket CO, Levine MM. Vaccines against enterotoxigenic Escherichia coli infections. Part ii: Live oral vaccines and subunit (purified fimbriae and toxin subunit) vaccines. New generation vaccines: Marcel Dekker, Inc, 1997:875-83.
6. Porter CK, Riddle MS, Tribble DR, Louis Bougeois A, McKenzie R, Isidean SD, Sebeny P, Savarino SJ. A systematic review of experimental infections with enterotoxigenic Escherichia coli (ETEC). Vaccine 2011;29:5869-85.
7. Ouwehand AC, ten Bruggencate SJ, Schonewille AJ, Alhoniemi E, Forssten SD, Bovee-Oudenhoven IM. Lactobacillus acidophilus supplementation in human subjects and their resistance to enterotoxigenic Escherichia coli infection. Br J Nutr 2014;111:465-73.
8. Ten Bruggencate SJ, Girard SA, Floris-Vollenbroek EG, Bhardwaj R, Tompkins TA. The effect of a multi-strain probiotic on the resistance toward Escherichia coli challenge in a randomized, placebo-controlled, double-blind intervention study. Eur J Clin Nutr 2015;69:385-91.
9. Harro C, Chakraborty S, Feller A, DeNearing B, Cage A, Ram M, Lundgren A, Svennerholm AM, Bourgeois AL, Walker RI, Sack DA. Refinement of a human challenge model for evaluation of enterotoxigenic Escherichia coli vaccines. Clin Vaccine Immunol 2011;18:1719-27.
10. Plackett RL, Burman JP. The Design of Optimum Multifactorial Experiments. Biometrika 1946;33:305–25.
11. Levine MM, Black RE, Clements ML, Young CR, Cheney CP, Schad P, al e. Prevention of enterotoxigenic Escherichia coli diarrheal infection in man by vaccines that stimulate anti-adhesion (anti-pilli) immunity. Attachment of organisms to the gut mucosa. Volume Boca Raton, FL; CRC Press, 1984:223-44.
12. Ten Bruggencate SJ, Frederiksen PD, Pedersen SM, Floris-Vollenbroek EG, Lucas-van de Bos E, van Hoffen E, Wejse PL. Dietary Milk-Fat-Globule Membrane Affects Resistance to Diarrheagenic Escherichia coli in Healthy Adults in a Randomized, Placebo-Controlled, Double-Blind Study. J Nutr 2016;146:249-55.
13. Heaton KW, Ghosh S, Braddon FE. How bad are the symptoms and bowel dysfunction of patients with the irritable bowel syndrome? A prospective, controlled study with emphasis on stool form. Gut 1991;32:73-9.
14. Svedlund J, Sjodin I, Dotevall G. GSRS--a clinical rating scale for gastrointestinal symptoms in patients with irritable bowel syndrome and peptic ulcer disease. Dig Dis Sci 1988;33:129-34.
15. Fleckenstein JM, Munson GM, Rasko DA. Enterotoxigenic Escherichia coli: Orchestrated host engagement. Gut Microbes 2013;4:392-6.
