# Supplementary material for: Low doses of diarrhoeagenic E. coli induce enhanced monocyte and mDC responses and prevent development of symptoms after homologous rechallenge
Source: PLoS One. 2023 Jan 6;18(1):e0279626. doi: 10.1371/journal.pone.0279626 (PMC9821474; doi:10.1371/journal.pone.0279626)
Supplement: Attachment — Submitted filename: Response to reviewers_MIRRE.pdf [file pone.0279626.s010.pdf]

Response to reviewers:

Reviewer #1: This manuscript reports a dose-response pilot study investigating which immunological factors are associated with this protection using healthy subjects. I have below comments and questions.

In Power calculation, it's not clear how n=6 was calculated. How big power was designed to achieve for what outcome or hypothesis test in this study? For this pilot study, power calculation is not necessary. Why do you use Plackett-Burman design? How did you use it? It is not related to this dose-response study. The cited reference 18 has nothing about Plackett-Burman design.

We would like to thank the reviewer for the time and effort to review our manuscript.

Reply: We acknowledge the fact that for a pilot study no "official" power analysis is required, however they believed it would be appropriate to follow such an approach especially so since in the present study a combination of a Design of Experiment (DOE) is linked with a "normal" efficacy study in which the impact of various doses of infectious *E. coli* was investigated on an immunological response. The design was set up by using the lowest dose (1E6) and the highest dose (1E10) as two factors in a 6-run DOE approach in which the other 3 doses were used to analyse the variance in the experiment (Box GEP, Hunter WG, Hunter JS. Statistics for experimenters. An Introduction to design, data analysis and model building. John Wiley&Sons, New York, US, 1978). Since this process was repeated 5 times (with the 5 doses applied in the study, taking the combination of two other doses as explanatory factor and the remaining ones as variance building blocks), a total number of 30 persons were included in the current experiment using the GEE analysis as method of choice. Therefore this approach was specifically applied to analyse the dose-dependent effect in the current study. We regret that reference 18 is not referring to the intended literature, being PLACKETT RL, BURMAN JP. THE DESIGN OF OPTIMUM MULTIFACTORIAL EXPERIMENTS. Biometrika 626 1946; 33(4): 305-325.For some reason the original reference was lost during amendment of the manuscript. Please find the publication listed again. Thank you for your attentiveness.

We have included aditional information in the "power calculation and statistical analysis" section in order to clarify the use of the DOE within the current analysis:

"In short, the highest and the lowest dose of infectious *E. coli* were used as two factors in a 6-run approach while the other 3 doses were used to analyse the overall variance in the outcome of the experiment. Since this process was repeated 5 times (with the 5 doses applied in the study, the combination of two other doses as explanatory factor and the remaining ones as variance building blocks), a total number of 30 persons was included in the current experiment."

In statistical analysis, it is not clear what stepwise approach was used and why it as needed. What nonlinear function of day and dose were included in GEE analysis? What software was used? Does "Fit of the model" mean "goodness of fit for the model" in your statement? What do you mean "when fit was significant"?

Reply: Please allow us to respond to this point issue by issue:

- Stepwise approach is used to evaluate the impact by the various confounding factors on the dependent parameter next to the fixed independent parameters. During each step that parameter with the most minimal explanation to the variation in the dependent parameter is taken away, that is to say, only when the p-value shows a non-significant contribution to the variation in outcome of the dependent parameter. At the same time the fit, or in your terms: goodness of fit of the model, is checked in such a way that the coverage of total variation in outcome is improved per step. During the last step a model is obtained with only significantly explaining parameters. This model can only be used when the goodness of fit is significant, showing that the model is indeed explaining the variation in outcome by using the remaining parameters significantly. By applying this methodology it is possible to identify the impact by every single parameter on the variation in outcome of the dependent parameter.

- Nonlinear function: quadratic (parabolic) and exponential, such as for time and dose of *E. coli*. During the stepwise approach, these factors will be removed when they demonstrate a non-significantly explaining contribution.
- Software used: Stata, College Station, TX, US.
- Fit of the model is equivalent to goodness of fit (of the model).
- "When fit was significant". Before saying anything on the contribution of (individual) parameters to the overall outcome, the model itself should be showing significance with respect to explaining the overall variation in outcome of the dependent parameter. In the event that this is not the case the individual parameters cannot demonstrate significance in any value since the overall covering of variation by the combined independent parameters is showing a non-significant nature.

Fig 4C and D may be analyzed using Cuzick's test for trend.

Reply: Thank you for this suggestion. As an alternative to the suggested test, we performed the one-way ANOVA post-test to check the linear trend using GraphPad Prism. In the 1st interval (Fig 4C), the slope of the line is positive but the linear trend was not conclusive (P-value = 0.07), most likely due to variation in the response of the highest dose. However, on the 2nd interval (Fig 4D), the slope of the line was negative and a clear linear trend was concluded (p-value = 0.0006). These results were added to the text and the fig. 4 legend.

In Correlation analysis, what statistical method was used for correlation?

Reply: The reviewer is right! this issue is not clearly described in the text. Throughout the manuscript, the correlation was evaluated via GEE analysis, taking into account the impact of confounding factors as there might be. We have included the following lines in the "power calculation and statistical analysis" section to clarify our approach:

"Correlation between parameters was investigated via General Estimating Equations (GEE) modelling including confounding parameters in a stepwise approach. When identified, a graphical check with the two parameters was done to verify their relationship."

In each figure legend, please add what statistical methods were used for analysis.

Reply: The requested data were added to the legend of Figures (3, 4, 5, 6, and 7) in the revised manuscript.

Reviewer #2: In their manuscript entitled: "Low doses of diarrheagenic E. coli induce enhanced monocyte and mDC responses and prevent development of symptoms after homologous rechallenge, the authors describe a challenge-rechallenge study in human volunteers to assess the protective immunity developed from low vs. high dose challenge of a diarrheagenic E. coli strain. They found that the symptoms and diarrhea induced by the primary challenge to be correlated with the challenge dose, all doses protected the volunteers from symptoms upon rechallenge, with stool frequency decreased in those who received the higher primary dose. Immunological markers of inflammation were greater during the primary challenge, with less inflammation during the secondary challenge, with the exception of dual IL-6 and TNF-alpha secreting PBMCs and monocytes, which increased after the second challenge. The manuscript was well written and easy to read.

I just have a few comments:

Some of the material in the methods section (Figure 1) is better in the results section.

We would like to thank the reviewer for the time and effort to review our manuscript.

Reply: Thank you for this comment. Indeed we agree with you but had to put the diagram in the Material and Methods section to meet journal requirements.

Why were only males included in the study? Why were females excluded?

Reply: Women were not eligible for this study because of the disadvantage of the female anatomy hindering fecal sample collection without urine contamination during acute infection. Another major issue is the interference of abdominal symptoms during their menstrual cycle.

The above statement was added to the manuscript in the material and methods section.

Minor comment:
Methods, p 13 line 133: if the dietary calcium intake was restricted, was it limited to <500 mg/day? Or is it correct as written?

Reply: The reviewer is right! The calcium intake was limited to less than 500mg/day. Therefore the symbol was adjusted in the revised version. Thank you for pointing this out.
