# Supplementary material for: Low doses of diarrhoeagenic E. coli induce enhanced monocyte and mDC responses and prevent development of symptoms after homologous rechallenge
Source: PLoS One. 2023 Jan 6;18(1):e0279626. doi: 10.1371/journal.pone.0279626 (PMC9821474; doi:10.1371/journal.pone.0279626)
Supplement: Attachment — Submitted filename: Response to reviewers_MIRRE_v2.0.pdf [file pone.0279626.s011.pdf]

Response to reviewers:

Reviewer #1: Please note, the reference list needs update. The reference 18 need to be replaced to Plackett-Burman's paper**.**

> **Reply:** Thank you for pointing out this issue. The reference was updated in the revised version and the mentioned reference is now number 19.

Reviewer #2: In their revised paper entitled "Low doses of diarrhoeagenic E. coli induce enhanced monocyte and mDC responses and prevent development of symptoms after homologous rechallenge" the authors have addressed the queries of the reviewer.

This reviewer is comfortable with the changes to the paper. I would remove the sentences explaining why this study does not include women. The reasoning of the authors is not consistent with other challenge studies conducted- there has been no difficulty collecting stool separately from women despite their anatomy, and most women can distinguish abdominal pain from a diarrheagenic infection vs. menstrual cramps.

> **Reply:** We would like to thank the reviewer for the comment. The statement was not initially there and it was added to the revised version 1.0. Based on the recommendations, we decided to remove the sentence.

Minor comment:
Results, Gastrointestinal symptom rating scale:
Line 261: "neither" should be "either"

> **Reply:** The word was corrected in the revised version of the manuscript.
