# Supplementary material for: Low doses of diarrhoeagenic E. coli induce enhanced monocyte and mDC responses and prevent development of symptoms after homologous rechallenge
Source: PLoS One. 2023 Jan 6;18(1):e0279626. doi: 10.1371/journal.pone.0279626 (PMC9821474; doi:10.1371/journal.pone.0279626)
Supplement: Attachment — Submitted filename: Porbahaie Review.docx [file pone.0279626.s009.docx]

In their manuscript entitled: “Low doses of diarrheagenic E. coli induce enhanced monocyte and mDC responses and prevent development of symptoms after homologous rechallenge, the authors describe a challenge-rechallenge study in human volunteers to assess the protective immunity developed from low vs. high dose challenge of a diarrheagenic E. coli strain. They found that the symptoms and diarrhea induced by the primary challenge to be correlated with the challenge dose, all doses protected the volunteers from symptoms upon rechallenge, with stool frequency decreased in those who received the higher primary dose. Immunological markers of inflammation were greater during the primary challenge, with less inflammation during the secondary challenge, with the exception of dual IL-6 and TNF-alpha secreting PBMCs and monocytes, which increased after the second challenge. The manuscript was well written and easy to read.

I just have a few comments:

Some of the material in the methods section (Figure 1) is better in the results section.

Why were only males included in the study? Why were females excluded?

Minor comment:

Methods, p 13 line 133: if the dietary calcium intake was restricted, was it limited to <500 mg/day? Or is it correct as written?
